# Near‐Infrared Switch‐Driven Macrophage Dynamically Reprogramming for Anti‐Infection and Tissue Healing on Polyetheretherketone Implants

**DOI:** 10.1002/advs.202512592

**Published:** 2025-11-04

**Authors:** Yinghao Wu, Shuaiqi Jiang, Jibing He, Ji Tan, Junjie Zhou, Zhipeng Zou, Jiaxing Wang, Jia Jiang, Xuanyong Liu, Xiaochun Peng

**Affiliations:** ^1^ Shanghai Sixth People's Hospital Affiliated to Shanghai Jiao Tong University School of Medicine Shanghai 200233 China; ^2^ State Key Laboratory of High Performance Ceramics Shanghai Institute of Ceramics Chinese Academy of Sciences Shanghai 200050 China; ^3^ Center of Materials Science and Optoelectronics Engineering University of Chinese Academy of Sciences Beijing 100049 China; ^4^ School of Chemistry and Materials Science Hangzhou Institute for Advanced Study University of Chinese Academy of Sciences Hangzhou 310024 China

**Keywords:** dynamic regulation, immune reprogramming, NIR responsive, antibacteria, tissue integration

## Abstract

High infection risk and poor tissue integration are major causes of percutaneous implant failure. Immune reprogramming is a promising strategy, but current implants rely predominantly on passive and static modulation of macrophages. Dynamically reprogramming macrophages based on physiological states to switch between antibacterial and tissue‐healing functions remains a challenge. Here, a novel polyetheretherketone (PEEK) surface is developed sequentially modified through sulfonation, hydrogen plasma immersion ion implantation (H‐PIII), and magnesium plasma immersion ion implantation (Mg‐PIII) to fabricate a Mg‐H‐SPEEK composite with a graphene‐like matrix embedded with MgO. This film simultaneously enables sustained Mg^2^⁺ release with near‐infrared (NIR) photothermal responsiveness for on‐demand immunomodulation. Under normal conditions, Mg^2^⁺ released from Mg‐H‐SPEEK can promote macrophage reprogramming toward the M2 phenotype through the TNF, JAK‐STAT, NF‐κB, and IL‐17 pathways to accelerate soft tissue repair. Upon NIR light exposure, photothermal stimulation enhances the expression of the Traf1 in macrophages via the TNF, FoxO, and JAK‐STAT signaling pathways to drive M1 reprogramming for bacterial phagocytosis. The dual‐mode system synergizes hyperthermia and immune phagocytosis for infection resistance while preserving pro‐healing functions, offering a smart strategy for percutaneous implants.

## Graphical abstract

This study develops a Mg‐H‐SPEEK implant with NIR‐switchable immunomodulatory properties. Under NIR, it promotes M1 macrophages for antibacterial action; without NIR, it induces M2 reprogramming to enhance tissue healing. This dual‐function material offers a smart strategy for reducing infection and improving integration in percutaneous implants.

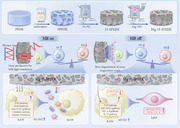

## 1 Introduction

The escalating global aging population has driven a marked increase in the prevalence of chronic diseases, including cardiovascular disease, diabetes, and osteoarthritis.^[^ ^1^ ^]^ This trend is compounded by the high incidence of accidental injuries, which has intensified the demand for effective implant solutions.^[^ ^2^ ^]^ Against this backdrop, the application of percutaneous implants has expanded significantly.^[^ ^3^ ^]^ Polyetheretherketone (PEEK) has garnered considerable attention in this context due to its excellent biocompatibility, mechanical properties, and chemical stability.^[^ ^4^ ^]^ However, the bioinertness of PEEK limits its integration with biological tissues and its ability to resist bacterial colonization, potentially leading to implant loosening and tissue infection, and ultimately resulting in implant failure.^[^ ^5^ ^]^ Current PEEK materials fall short in terms of antibacterial properties and tissue integration capabilities, failing to meet the growing clinical demands.^[^ ^6^ ^]^ Given these challenges, surface modification of PEEK is essential to enhance tissue integration and improve antibacterial properties.

In the biomedical field, the immune system plays a pivotal role in both anti‐infection and tissue healing processes, particularly through macrophage reprogramming.^[^ ^7^ ^]^ Macrophages exhibit plasticity and can be reprogrammed into different phenotypes in response to microenvironmental signals.^[^ ^8^ ^]^ M1 macrophages primarily produce pro‐inflammatory cytokines (e.g., IL‐1β, IL‐6, and TNF‐α) to eliminate pathogens and initiate inflammatory responses.^[^ ^9^ ^]^ However, persistent M1 activation can cause excessive inflammation, delaying tissue healing and leading to chronic inflammatory states.^[^ ^10^ ^]^ In contrast, M2 macrophages are associated with tissue healing and remodeling. They secrete anti‐inflammatory cytokines (e.g., IL‐10 and TGF‐β) that help mitigate inflammation and promote tissue healing.^[^ ^11^ ^]^ M2 macrophages also facilitate collagen deposition and extracellular matrix remodeling, creating a favorable microenvironment for tissue healing and regeneration.^[^ ^12^ ^]^ The balance between M1 and M2 macrophages is crucial for achieving both antibacterial and tissue healing objectives.^[^ ^11^ ^]^ Existing PEEK implants predominantly employ passive and static methods to modulate macrophages, which makes it difficult to dynamically regulate the state of macrophages according to different physiological states, and thereby flexibly switch between antibacterial and tissue healing functions.

In recent years, near‐infrared (NIR) photothermal therapy has emerged as a promising biomedical technology that can modulate macrophage phenotype reprogramming, thereby playing a significant role in tissue healing and immune regulation.^[^ ^13^ ^]^ Studies have shown that increasing the surface temperature of materials can enhance macrophage TNF expression, thereby improving bacterial phagocytosis and antibacterial efficacy.^[^ ^14^ ^]^ However, excessive light power can cause abnormal temperature increases in surrounding tissues, adversely affecting the healing process.^[^ ^15^ ^]^ To address this issue, researchers have explored various materials to improve photothermal conversion efficiency.^[^ ^16^ ^]^ Planar light‐absorbing materials, with their singular light absorption and reflection characteristics, are insufficient for efficient energy conversion.^[^ ^17^ ^]^ In contrast, porous materials can significantly enhance photothermal efficiency by reflecting and absorbing photons multiple times.^[^ ^18^ ^]^ Magnesium ions (Mg^2^⁺) are abundant in the human body and essential for bone tissue and tissue repair.^[^ ^19^ ^]^ Mg^2^⁺ also modulates the immune microenvironment by promoting M2 macrophages programmation, thereby creating a favorable environment for both bone healing and tissue repair.^[^ ^20^ ^]^ Therefore, a material with a porous structure that can induce M1 macrophages reprogramming for antibacterial effects under NIR stimulation and M2 reprogramming for tissue healing through Mg^2^⁺ release in a normal state would meet clinical requirements.

Despite significant advancements in PEEK surface modification, the chemical inertness of PEEK still limits the effectiveness of most methods.^[^ ^21^ ^]^ Thus, achieving efficient and stable surface modification remains a critical challenge. Plasma technology offers a potential solution. Plasma immersion ion implantation (PIII) is a technique that uses negative high voltage to implant gases or metals into the surface of materials in a plasma state.^[^ ^22^ ^]^ High‐energy plasma implantation can form a graphitic film on the polymer surface, with hydrogen plasma implantation yielding better graphitization effects.^[^ ^23^ ^]^ Additionally, metals and metal oxides can be ion‐implanted or deposited on a strongly bonded substrate.^[^ ^24^ ^]^ Sulfonated polyetheretherketone (SPEEK) is a material obtained by treating the surface of PEEK with concentrated sulfuric acid. Its surface forms a porous structure due to the electrostatic repulsion between sulfonic acid groups.^[^ ^25^ ^]^

**Scheme 1:**
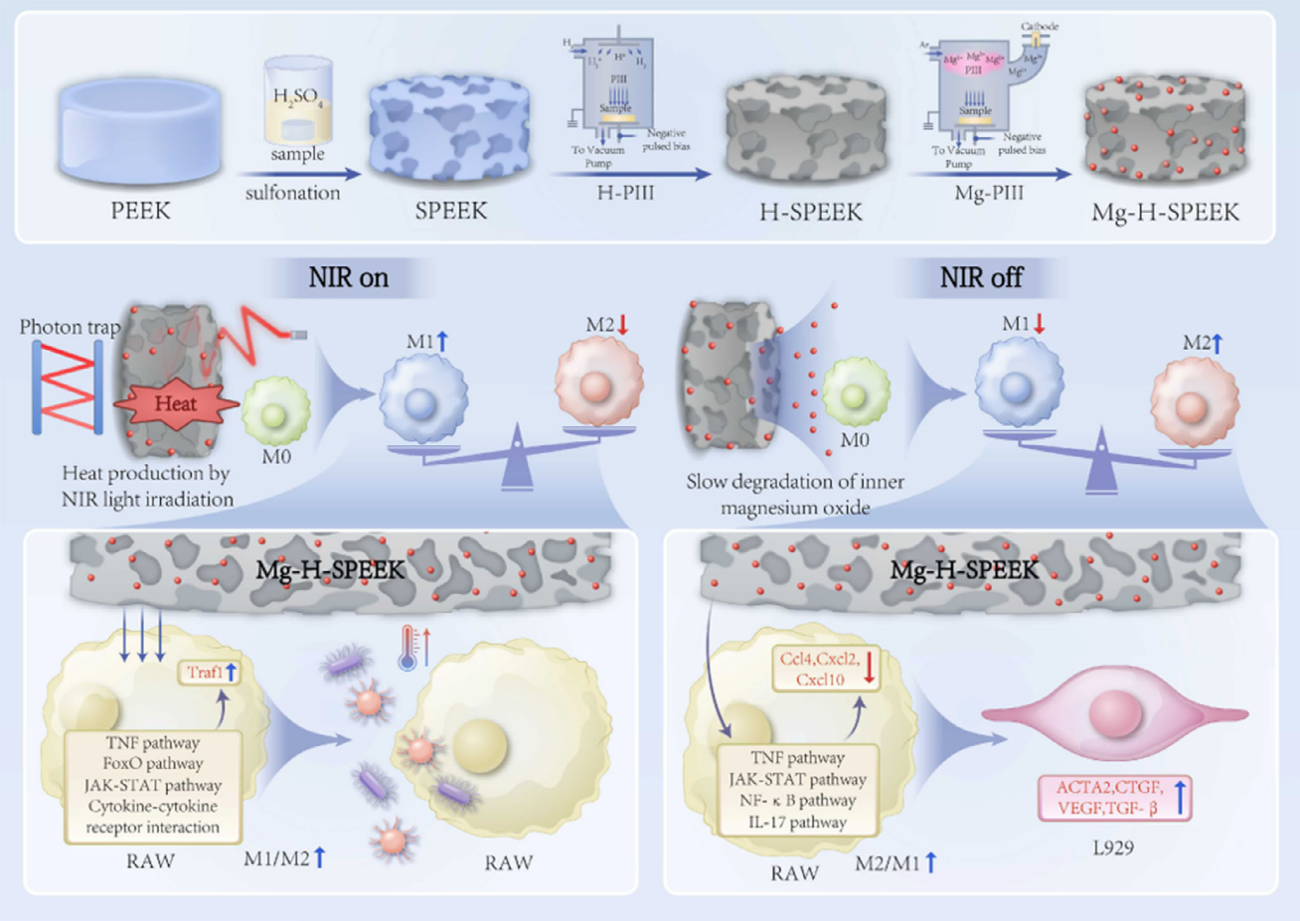
Synthesis of Mg‐H‐SPEEK and the modulatory effects of NIR irradiation on the reprogramming of RAW264.7 macrophages. The process begins with the fabrication of SPEEK from PEEK, followed by hydrogen plasma immersion ion implantation (H‐PIII) on the SPEEK surface to create a graphene‐like film with photon trapping properties. Thereafter, Magnesium ions (Mg^2^⁺) are incorporated via magnesium plasma immersion ion implantation (Mg‐PIII). Upon exposure to near‐infrared (NIR) radiation, the Mg‐H‐SPEEK leverages its photothermal properties to absorb NIR photons, thereby generating heat. This heat triggers the upregulation of Traf1 expression through various signaling pathways, including NF‐κB, FoxO, JAK‐STAT, and Cytokine‐cytokine receptor interactions. Consequently, this promotes the reprogramming of RAW264.7 macrophages toward the M1 phenotype and enhances their phagocytic capacity. In contrast, under conditions devoid of NIR irradiation, the Mg^2^⁺ released by Mg‐H‐SPEEK downregulate the expression of Ccl4, Cxcl2, and Cxcl10 via pathways such as TNF, JAK‐STAT, NF‐κB, and IL‐17. This shift facilitates the reprogramming of RAW264.7 macrophages toward the M2 phenotype, which in turn upregulates the expression of ACTA2, CTGF, VEGF, and TGF‐β in L929 fibroblasts, thereby augmenting their tissue healing capabilities.

Based on this background, we designed a novel material—Mg‐H‐SPEEK—by sulfonating PEEK and then using a two‐step plasma immersion ion implantation method to construct a porous graphitic film embedded with magnesium oxide (MgO) nanoparticles on the SPEEK surface. This material can dynamically regulate immune responses by reprogramming macrophages, thereby promoting tissue healing (Scheme **1**). Under NIR irradiation, Mg‐H‐SPEEK significantly enhances macrophage phagocytic capacity, effectively engulfing bacteria and achieving synergistic antibacterial effects through photothermal action. When NIR irradiation ceases, Mg‐H‐SPEEK reprograms macrophages to the M2 phenotype, facilitating tissue healing. This dynamic regulatory capability enables Mg‐H‐SPEEK to rapidly eliminate pathogens during the initial infection phase and promote tissue healing during the healing phase.

In this study, we developed Mg‐H‐SPEEK and validated its superior antibacterial, immune‐regulatory, and tissue healing properties through in vitro experiments. Using RNA sequencing (RNA‐seq), we explored the mechanisms underlying NIR‐induced phagocytosis and immune reprogramming, as well as the immune regulatory mechanisms of Mg‐H‐SPEEK. Finally, we evaluated its anti‐infection, immune‐regulatory, and tissue healing effects using percutaneous infection and implantation models. We hypothesize that Mg‐H‐SPEEK can dynamically regulate macrophage reprogramming to enhance antibacterial and tissue healing functions, thereby improving the clinical performance of percutaneous implants.

## 2 Results and Discussion

### 2.1 Surface Morphologies and Characterization of Samples

**Figure 1:**
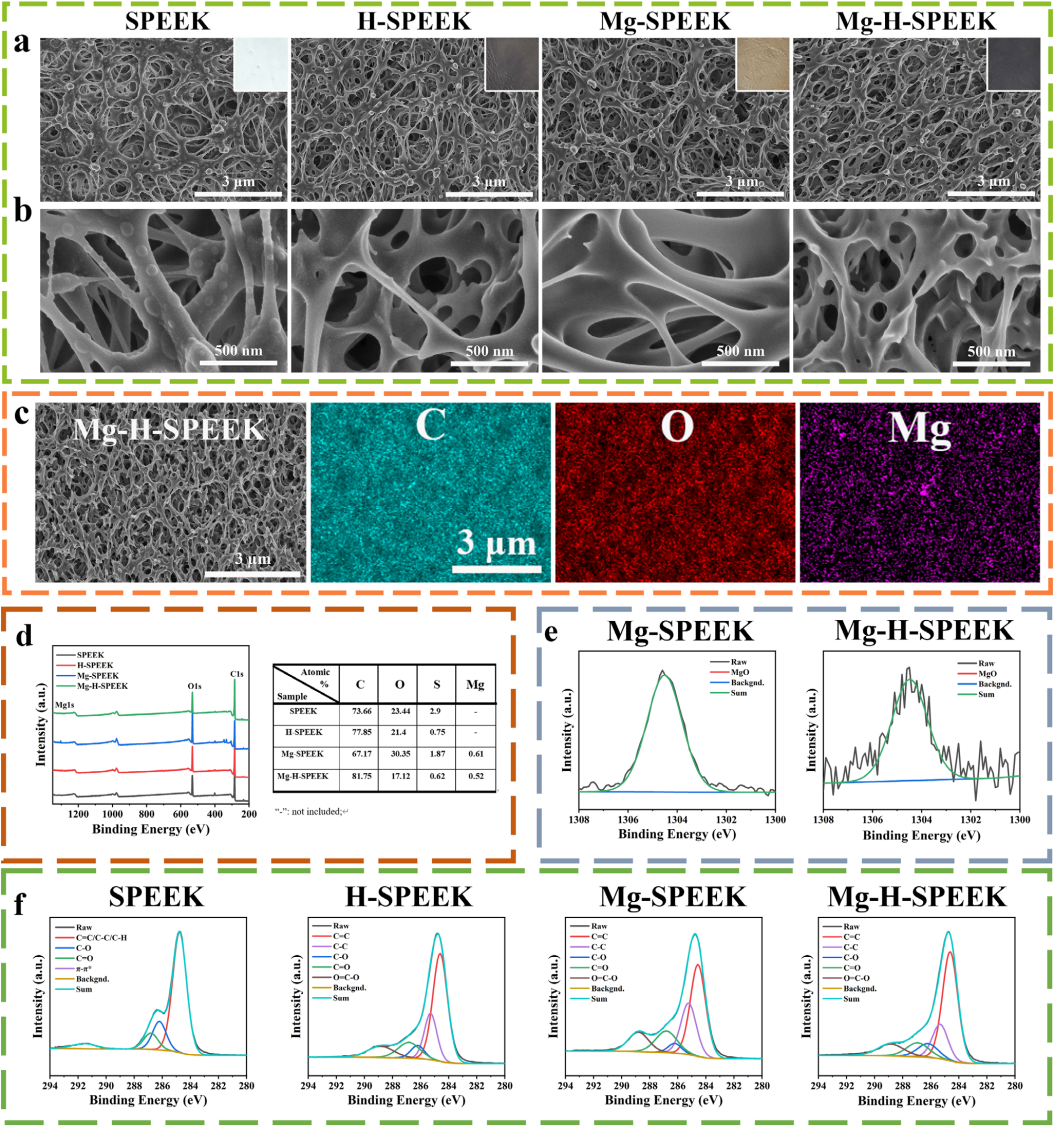
Surface morphologies and component analysis of PEEK, H‐SPEEK, Mg‐SPEEK, and Mg‐H‐SPEEK samples. a) Optical and SEM images. b) High magnification SEM images. c) EDS spectra of Mg‐H‐SPEEK. d) XPS survey spectra and elemental content of the sample surfaces. e) High‐resolution XPS spectra of the Mg 1s for the Mg‐SPEEK and Mg‐H‐SPEEK. f) High‐resolution XPS spectra of the C 1s region.

The Scanning Electron Microscopy (SEM) images (**Figure** **1**a,b) visually depict the surface morphology of the polymer electrolyte samples. The surface of SPEEK exhibits a uniform 3D porous network structure. After Plasma Immersion Ion Implantation (PIII) treatment, etching marks from the plasma are evident on the porous framework of the sample surface, yet the main structure remains unchanged. The Energy Dispersive Spectroscopy (EDS) images (Figure 1c) reveal that following Mg‐PIII treatment, Magnesium ions (Mg^2^⁺) elements are uniformly distributed across the sample surface. The X‐ray Photoelectron Spectroscopy (XPS) survey spectra (Figure 1d) indicate that Mg^2^⁺ elements are only present on the surfaces of Mg‐SPEEK and Mg‐H‐SPEEK samples, a finding further corroborated by elemental analysis results. Intriguingly, compared with SPEEK, the surfaces of H‐SPEEK and Mg‐H‐SPEEK have a higher content of carbon elements, which is in accordance with the results from our previous work.^[^ ^26^ ^]^ The high‐resolution spectra of Mg 1s for the Mg‐SPEEK and Mg‐H‐SPEEK surfaces are shown in Figure 1e, with their binding energy peaks located at 1303.9 eV, corresponding to the binding energy of divalent Mg^2^⁺ in MgO. The high‐resolution XPS spectra of the C 1s region (Figure 1f) elucidate the various carbon environments within the samples, shedding light on the chemical structure of the polymer electrolytes. Peak fitting analysis of the C 1s spectra was conducted to identify the chemical functional groups. The C 1s peak was primarily deconvoluted into five sub‐peaks. The peak at 284.6 eV originates from carbon–carbon double bonds (C═C), the peak at 285.3 eV from carbon–carbon single bonds (C─C), and the peaks at 286.2, 286.8, and 288.4 eV correspond to ether bonds (C─O), carbonyl groups (C═O), and carboxyl groups (O ─C═O), respectively. Compared with SPEEK, the surfaces of H‐SPEEK and Mg‐H‐SPEEK have a higher relative content of sp2‐hybridized carbon. The carbon elements in the graphitic‐like coating primarily exist in the form of sp2‐hybridized carbon, and a higher relative content indicates a greater propensity for the formation of a graphitic‐like coating on the material surface.

### 2.2 Physicochemical Properties of Samples

**Figure 2:**
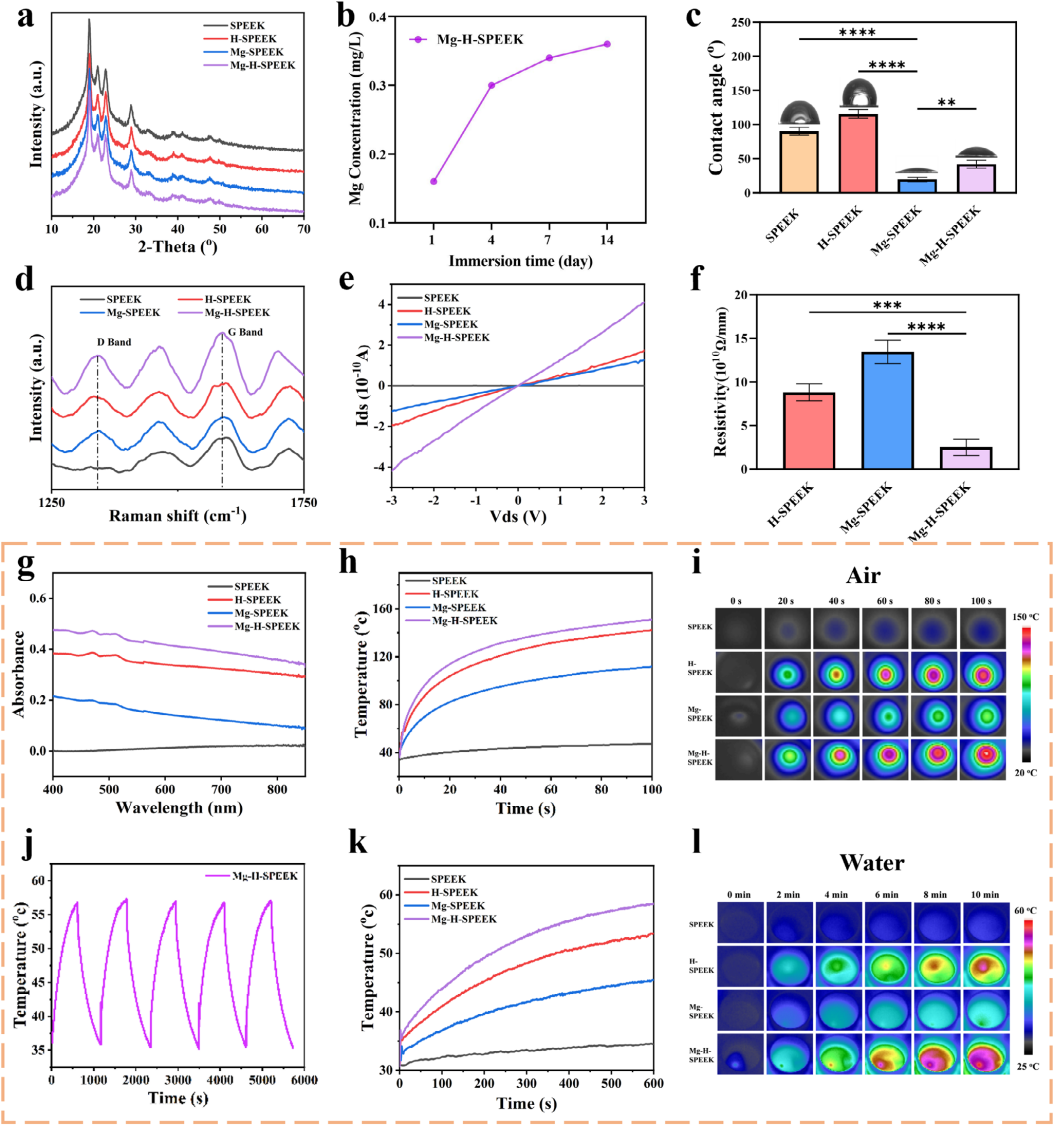
Comprehensive characterization of SPEEK, H‐SPEEK, Mg‐SPEEK, and Mg‐H‐SPEEK materials. a) XRD spectra. b) Cumulative release results of Mg^2^⁺ from Mg‐H‐SPEEK. c) Contact angle measurements. d) Raman spectra. e) Current‐voltage (*I–V*) curves and f) corresponding calculation results. g) UV–vis spectra. h) Temperature change and i) Infrared thermal images of different samples under 808 nm NIR at 0.8 W cm^−2^ in air. j) Temperature variations of Mg‐H‐SPEEK samples for 5 on/off cycles in 1 mL ultrapure water. k) Temperature change and l) Infrared thermal images of different samples under 808 nm NIR at 0.8 W cm^−2^ in 1 mL ultrapure water. ^**^ *p* < 0.01; ^***^ *p* < 0.001; ^****^ *p* < 0.0001.

**Figure** **2**a presents the X‐ray diffraction (XRD) patterns of SPEEK, H‐SPEEK, Mg‐SPEEK, and Mg‐H‐SPEEK. The XRD characteristic peak intensities of the three PIII‐treated samples were reduced to varying degrees, indicating that the ion implantation process caused some damage to the crystalline structure of the material surface. Figure 2b shows the ion release results of Mg‐H‐SPEEK, demonstrating that the material can continuously release Mg^2^⁺ over a period of 14 days. Furthermore, Figure S4a (Supporting Information) presents the Mg^2^⁺ release profiles across a spectrum of pH levels (6.5, 7.4, and 8.5), and Figure S4b (Supporting Information) shows the Mg^2^⁺ release under various NIR irradiation durations (0, 3, 5, and 7 min). Collectively, these graphs indicate that Mg^2^⁺ ion release is fairly stable and is not significantly influenced by changes in pH or the temperature conditions resulting from NIR irradiation, implying a robust Mg^2^⁺ release kinetics that is largely independent of these variables.

The contact angles of the surfaces of SPEEK, H‐SPEEK, Mg‐SPEEK, and Mg‐H‐SPEEK were measured to be 90.2°, 115.7°, 19.6°, and 42.2°, respectively. The enhanced hydrophobicity of the H‐SPEEK surface is consistent with the XPS results showing reduced oxygen‐containing functional groups (Figure 2c). The increased hydrophilicity of the Mg‐SPEEK and Mg‐H‐SPEEK surfaces may be attributed to the increased oxygen‐containing functional groups following Mg‐PIII treatment. Figure S2 (Supporting Information) displays the FTIR spectra of all samples, with the characteristic peak of the carbonyl group appearing at 1710 cm^−1^, suggesting changes in the surface structure. Figure 2d shows the Raman spectra of the samples. The absorption peak at 1580 cm^−1^ (G peak) is characteristic of the sp2 carbon structure. Since PEEK molecular chains contain a large number of benzene rings, the G peak is present in all four samples. The absorption peak at 1350 cm^−1^ (D peak) represents the disordered carbon structure, indicating defects in the graphite structure. Compared with SPEEK, the D peak is present in H‐SPEEK, Mg‐SPEEK, and Mg‐H‐SPEEK, suggesting the formation of a graphitic‐like structure on their surfaces. Figure 2e,f shows the surface electrical conductivity results of the samples. Increased surface graphitization enhances the free movement area of electrons, thereby improving surface electrical conductivity. The conductivity measurement device could not detect any electrical signal from SPEEK within the voltage range of −3–3 V, indicating its high surface resistivity and insulating nature. Mg‐H‐SPEEK exhibited the lowest resistivity and highest electrical conductivity, indicating the highest degree of graphitization. Figure 2g shows the UV–vis absorption spectra of different samples. The variation in graphitization degree implies changes in the conjugated system size, which in turn affects surface absorbance. After PIII treatment, all samples exhibited enhanced absorbance in the visible light region, indicating an extended conjugated system. Mg‐H‐SPEEK showed the highest absorbance. To further characterize the differences in surface graphitization, we considered that the porous structure on the surface can enhance light absorption by acting as a photonic trap, enhancing light absorption. We validated this through experiments by subjecting PEEK and SPEEK to the same H‐PIII treatment to confirm the enhanced light absorption effect due to the 3D porous structure. As shown in Figure S3a (Supporting Information), the main morphology of the materials was not destroyed after PIII treatment. Compared with H‐P, H‐SP exhibited a 3D porous structure with a larger light absorption area. Figure S3b,c (Supporting Information) show the light absorption and photothermal effects of PEEK, SPEEK, H‐P, and H‐SP. H‐SP, with its 3D porous structure acting as a photonic trap, exhibited the strongest light absorption and photothermal effects. Generally, materials with higher porosity have a larger specific surface area, facilitating light absorption. By adjusting the sulfonation time, we constructed a series of surfaces with different pore structures on PEEK and explored the relationship between pore structure and light absorption. As shown in Figure S4a (Supporting Information), with the extension of sulfonation time, the porosity and pore size of the material surface increased. Figure S4b,c (Supporting Information) shows the light absorption and photothermal effects of the samples, with H‐SP‐12 exhibiting the best performance. The photothermal effect of SPEEK, H‐SPEEK, Mg‐SPEEK, and Mg‐H‐SPEEK under 808 nm NIR irradiation was investigated. First, the temperature changes and infrared thermal images of each group were examined under NIR irradiation at a power density of 0.8 W cm^−2^ in air for 100s (Figure 2h,i). The maximum temperatures of the SPEEK, H‐SPEEK, Mg‐SPEEK, and Mg‐H‐SPEEK samples were 47.2, 111.4, 141.9, and 151.1 °C, respectively. This was further verified by real‐time thermal imaging. Subsequently, the photothermal performance and photothermal cycling performance of the samples were tested in aqueous solution conditions. Under the same power density, the temperature changes and infrared thermal images of each group in 1 mL ultrapure water were tested within 10 min (Figure 2k,l). A light intensity of 0.8 W cm^−2^ caused the external temperature of Mg‐H‐SPEEK to rise to ≈58.4 °C after 10 min, indicating its optimal photothermal performance. H‐SPEEK exhibited the second‐best performance, with a temperature increase to 53.2 °C within 10 min. SPEEK and Mg‐SPEEK had relatively lower photothermal conversion capabilities. Finally, the photothermal cycling curve of the Mg‐H‐SPEEK sample was tested. According to the results shown in Figure 2j, the sample exhibited good photothermal cycling performance.

### 2.3 Antibacterial Properties of SPEEK and Mg‐H‐SPEEK under NIR Irradiation

**Figure 3:**
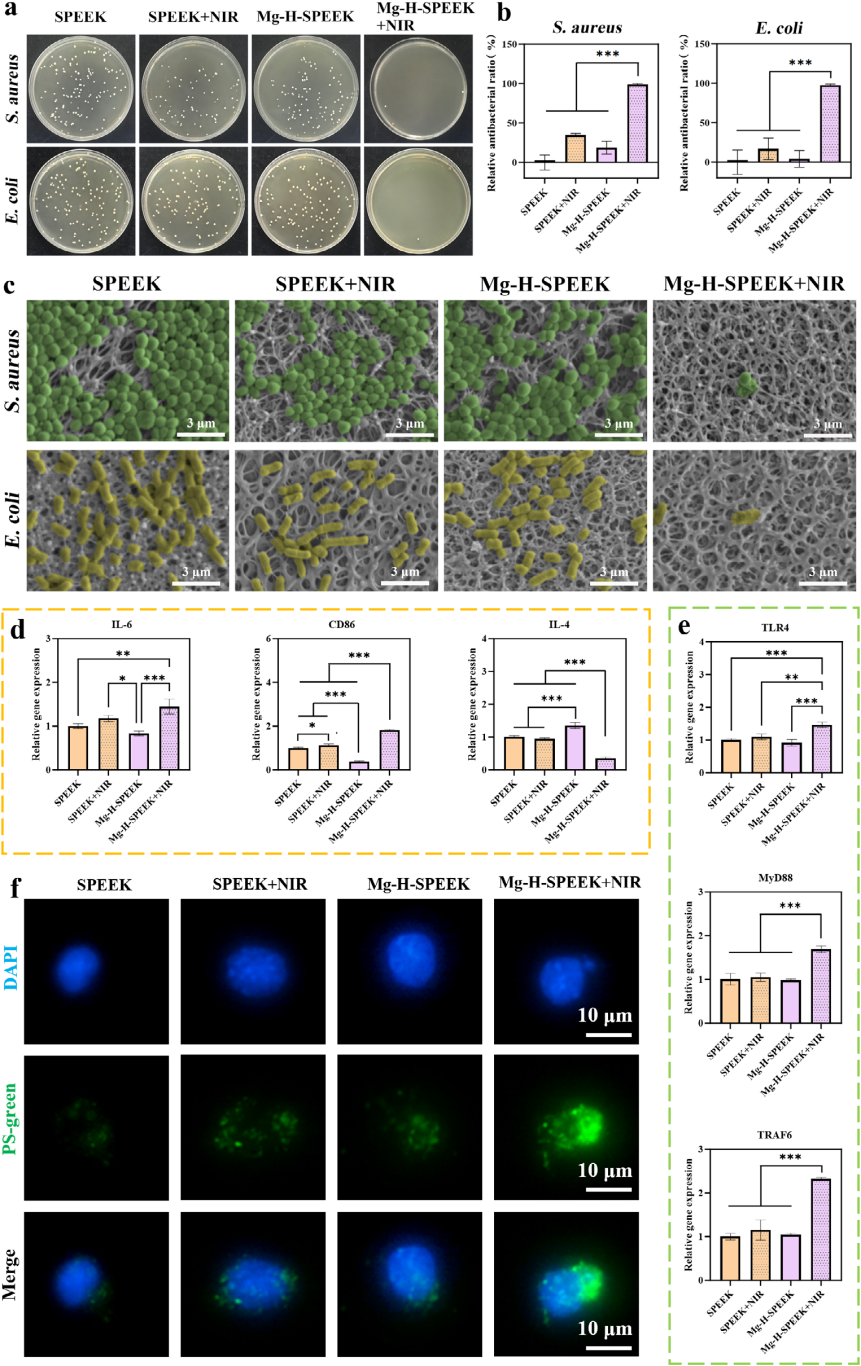
Antibacterial effects and macrophage immune responses, and phagocytosis efficiency of SPEEK and Mg‐H‐SPEEK under NIR Irradiation. a) Photographs of *S. aureus* and *E. coli* colony plates on various material surfaces, accompanied by b) the corresponding relative antibacterial ratios. c) SEM images depicting the surface topography of each material group, with green indicating the presence of *S. aureus* and yellow indicating *E. coli*. d) mRNA expression levels of M1 macrophage markers (IL‐6, CD86) and M2 macrophage markers (IL‐4) in RAW264.7 macrophages. e) mRNA expression levels of phagocytosis‐related markers (TLR4, MyD88, TRAF6) in RAW264.7 macrophages. f) Fluorescence microscopy images of RAW264.7 macrophages engulfing PS microspheres. ^**^ *p* < 0.01; ^***^ *p* < 0.001.

Here, the bacterial removal capabilities of SPEEK, SPEEK+NIR, Mg‐H‐SPEEK, and Mg‐H‐SPEEK+NIR were evaluated. **Figure** **3**a displays the colony plate images of Staphylococcus aureus (*S. aureus*) and Escherichia coli (*E. coli*) on SPEEK, SPEEK+NIR, Mg‐H‐SPEEK, and Mg‐H‐SPEEK+NIR. Compared with SPEEK, the number of bacterial colonies on the Mg‐H‐SPEEK surface was reduced, likely due to the weak alkaline microenvironment generated on the surface during the degradation of MgO. After NIR treatment, the number of bacterial colonies on the Mg‐H‐SPEEK+NIR surface significantly decreased compared to SPEEK+NIR, attributed to the photothermal effect of the film. Further quantitative analysis (Figure 3b) revealed that, compared to SPEEK, the antibacterial rates of SPEEK+NIR, Mg‐H‐SPEEK, and Mg‐H‐SPEEK+NIR against *E.coli* were 17.0%, 4.2%, and 97.6%, respectively, while those against *S. aureus* were 34.7%, 18.7%, and 98.6%, respectively. The morphology of *S. aureus* and *E. coli* on the surfaces of SPEEK, SPEEK+NIR, Mg‐H‐SPEEK, and Mg‐H‐SPEEK+NIR was observed using SEM (Figure 3c). The morphology of both types of bacteria on the SPEEK, SPEEK+NIR, and Mg‐H‐SPEEK surfaces was relatively intact, with typical complete spherical and rod‐shaped forms for *S. aureus* and *E. coli*, respectively. The number of bacteria on the Mg‐H‐SPEEK+NIR surface was the lowest, with evident bacterial membrane degeneration. These results indicate that Mg‐H‐SPEEK exhibits excellent antibacterial efficacy against both types of bacteria under NIR irradiation.

### 2.4 Immune and Phagocytic Responses of Macrophages to SPEEK and Mg‐H‐SPEEK under NIR Irradiation

In addition to its antibacterial properties, the immunomodulatory and phagocytic effects of the samples on RAW264.7 macrophages were evaluated by detecting the expression levels of immune‐related and phagocytosis‐related genes in RAW264.7 macrophages via qPCR, and by assessing the phagocytic capacity for fluorescent polystyrene (PS) microspheres on the sample surfaces using fluorescence detection. Figure 3d shows the mRNA expression levels of M1 (IL‐6, CD86) and M2 (IL‐4) markers in RAW264.7 macrophages. The results indicate that NIR irradiation enhanced the expression of M1 markers IL‐6 and CD86 in both SPEEK and Mg‐H‐SPEEK groups, with the most significant upregulation observed in the Mg‐H‐SPEEK + NIR group. Conversely, in the Mg‐H‐SPEEK group subjected to NIR irradiation, the expression of the M2 marker IL‐4 was significantly downregulated compared to the untreated group. This change was not observed in the SPEEK group. These findings suggest that NIR irradiation promotes the conversion of RAW264.7 macrophages to the M1 phenotype on the Mg‐H‐SPEEK surface. Figure 3e reveals the mRNA expression levels of phagocytosis‐related markers (TLR4, MyD88, TRAF6). The expression levels in the Mg‐H‐SPEEK+NIR group were significantly higher than those in the other groups, indicating a substantial enhancement in phagocytic capacity. This finding was further confirmed by fluorescence microscopy images (Figure 3f), which showed that RAW264.7 macrophages in the Mg‐H‐SPEEK+NIR group had the largest fluorescent area of engulfed PS microspheres, intuitively demonstrating their superior phagocytic ability compared to other groups.

In summary, Mg‐H‐SPEEK exhibits excellent photothermal effects, which can kill bacteria. Moreover, NIR stimulation can enhance the M1 reprogramming capacity and phagocytic activity of RAW264.7 macrophages, thereby improving the phagocytic efficacy against bacteria.

### 2.5 Analysis of Inflammation and Soft Tissue Healing in Single‐Culture and Co‐Culture Systems

**Figure 4:**
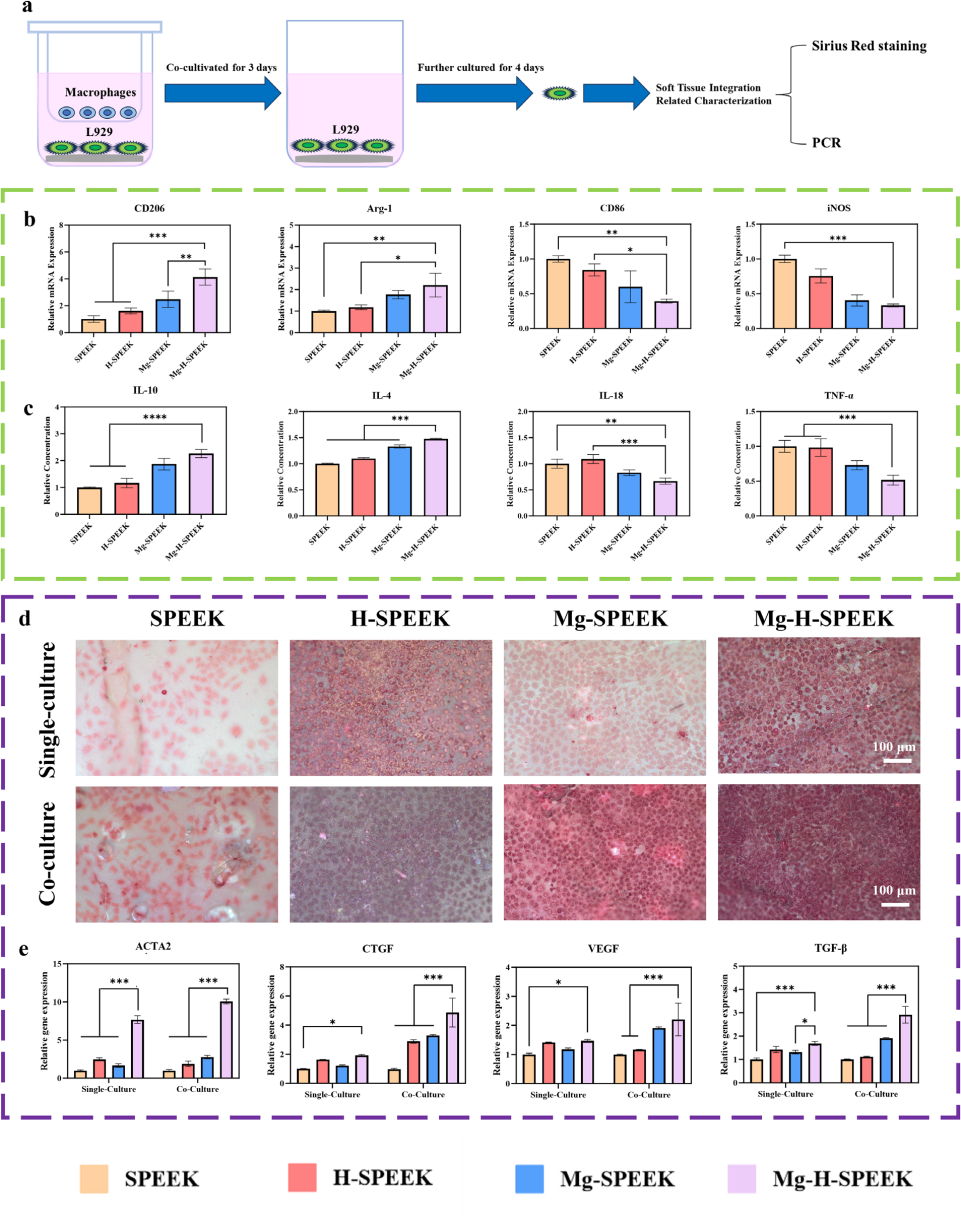
Inflammation and soft tissue analysis in the Single‐Culture and Co‐Culture system. a) Schematic diagram of Co‐Culture of L929 fibroblasts and RAW264.7 macrophages. b) mRNA expression levels of inflammatory markers CD206, Arg‐1, CD86, and iNOS in RAW264.7 macrophages were measured in both Single‐Culture and Co‐Culture systems. c) Quantitative ELISA results for inflammatory cytokines in RAW264.7 macrophages are shown. d) Sirius Red staining was performed to assess collagen secretion by L929 fibroblasts in Single‐Culture and Co‐Culture systems. e) mRNA expression levels of soft tissue‐promoting factors ACTA2, CTGF, VEGF, and TGF‐β in L929 fibroblasts were evaluated in both Single‐Culture and Co‐Culture systems. Statistical significance is indicated by ^*^ *p* < 0.05, ^**^ *p* < 0.01, ^***^ *p* < 0.001, ^****^ *p* < 0.0001.

To investigate the effects of SPEEK, H‐SPEEK, Mg‐SPEEK, and Mg‐H‐SPEEK on inflammation and soft tissue healing capabilities, we designed a series of experiments. Initially, RAW264.7 macrophages were cultured individually (Single‐Culture) to assess the regulatory effects of these materials on inflammation. Additionally, to more accurately simulate the intercellular interactions within the body, we employed the experimental design shown in **Figure** **4**a, co‐culturing L929 fibroblasts with RAW264.7 macrophages (Co‐Culture). In this Co‐Culture system, L929 fibroblasts and RAW264.7 macrophages were seeded in the lower and upper chambers, respectively. After 3 days of Co‐Culture, the RAW264.7 macrophages in the upper chamber were removed to study the effects of the materials on L929 fibroblasts under both Single‐Culture and Co‐Culture conditions.

When evaluating the effects of different materials on the proliferation of RAW264.7 macrophages and L929 fibroblasts, we found that compared to SPEEK, H‐SPEEK, Mg‐SPEEK, and Mg‐H‐SPEEK all enhanced the proliferative capacity of RAW264.7 macrophages and L929 fibroblasts, although the increase was modest (Figures S5 and S6, Supporting Information). This result indicates that these materials exhibited no significant cytotoxicity in terms of cell compatibility, providing a foundation for further research.

Regarding inflammation, our results demonstrated that compared to H‐SPEEK and SPEEK surfaces, Mg‐H‐SPEEK and Mg‐SPEEK surfaces significantly upregulated the mRNA expression of M2 macrophage markers CD206 and Arg‐1 (Figure 4b), suggesting that Mg‐H‐SPEEK and Mg‐SPEEK promote macrophage reprogramming toward the M2 phenotype. Conversely, the expression of M1 macrophage markers CD86 and iNOS was lower on Mg‐H‐SPEEK and Mg‐SPEEK surfaces (Figure 4b), consistent with the results shown in Figure 3d, further confirming the ability of Mg‐H‐SPEEK and Mg‐SPEEK surfaces to inhibit M1 macrophages. To further validate these trends, we quantitatively measured the levels of anti‐inflammatory cytokines (IL‐10, IL‐4) and pro‐inflammatory cytokines (IL‐18, TNF‐α) using ELISA. The ELISA results shown in Figure 4c were consistent with the qPCR data, further confirming the positive regulatory effects of Mg‐H‐SPEEK and Mg‐SPEEK on inflammatory responses.

Sirius Red staining was employed to elucidate the collagen secretion by L929 fibroblasts, which is commonly used to assess extracellular matrix synthesis.^[^ ^27^ ^]^ In the Single‐Culture system, Mg‐H‐SPEEK and H‐SPEEK exhibited higher staining intensity (Figure 4d), which was further supported by quantitative analysis (Figure S7, Supporting Information), indicating their superior ability to promote collagen secretion. In the Co‐Culture system, Sirius Red staining intensity on Mg‐H‐SPEEK and Mg‐SPEEK surfaces significantly increased compared to the other two groups (Figure 4d), a finding corroborated by quantitative analysis (Figure S7, Supporting Information). This suggests that the Co‐Culture system further enhanced the ability of Mg‐H‐SPEEK and Mg‐SPEEK to reprogram macrophages toward the M2 phenotype, thereby significantly promoting collagen secretion. Additionally, we analyzed the mRNA expression levels of soft tissue healing factors, such as ACTA2, CTGF, VEGF, and TGF‐β, using qPCR. In the Single‐Culture system, higher expression was observed on Mg‐H‐SPEEK and H‐SPEEK surfaces (Figure 4e). In the Co‐Culture system, the expression of these genes was significantly enhanced on Mg‐H‐SPEEK and Mg‐SPEEK surfaces, surpassing that of the other two groups (Figure 4e). These findings, consistent with the Sirius Red staining results, further confirmed the soft tissue promotion capabilities of Mg‐H‐SPEEK and Mg‐SPEEK. Finally, scratch assays on L929 fibroblasts further supported the soft tissue healing capabilities of Mg‐H‐SPEEK and Mg‐SPEEK. In the Co‐Culture system, Mg‐H‐SPEEK and Mg‐SPEEK exhibited higher cell migration capacity compared to their counterparts in the Single‐Culture system (Figure S8, Supporting Information). Collectively, these results indicate that Mg‐H‐SPEEK and Mg‐SPEEK significantly promote the reprogramming of RAW264.7 macrophages to the M2 phenotype, thereby stimulating L929 fibroblasts to better facilitate soft tissue healing.

### 2.6 RNA‐seq Analysis of Mg‐H‐SPEEK: NIR‐Induced Effects and Anti‐Inflammatory Mechanisms

**Figure 5:**
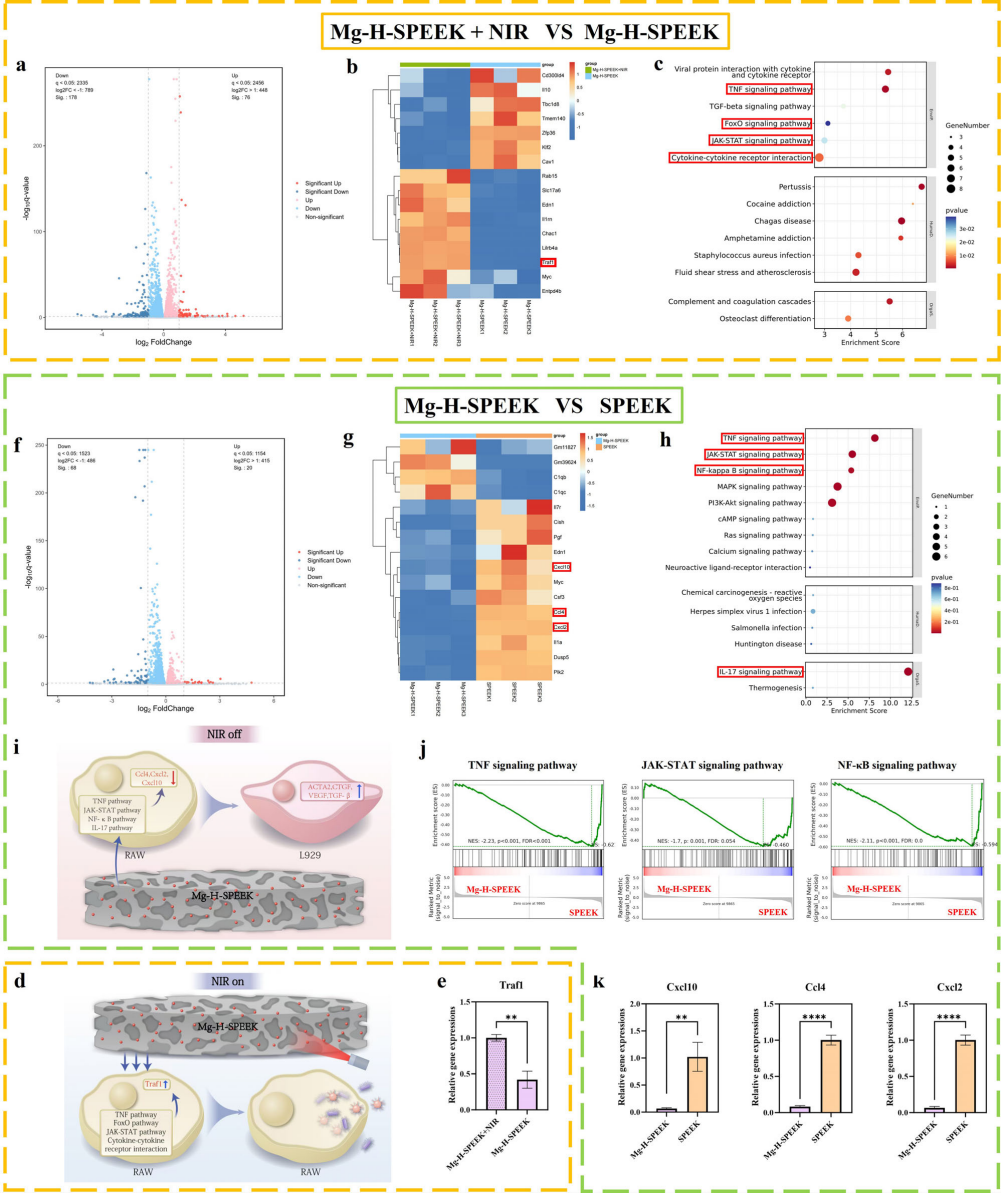
RNA‐seq analysis of Mg‐H‐SPEEK versus SPEEK and Mg‐H‐SPEEK+NIR versus Mg‐H‐SPEEK. a) Volcano plot of DEGs between Mg‐H‐SPEEK+NIR versus Mg‐H‐SPEEK. b) Heatmap of DEGs between Mg‐H‐SPEEK+NIR versus Mg‐H‐SPEEK. c) Enriched pathways by KEGG analysis for Mg‐H‐SPEEK+NIR versus Mg‐H‐SPEEK. d) Schematic diagram of the mechanism of NIR‐enhanced phagocytosis. e) Relative expression levels of Traf1 in Mg‐H‐SPEEK+NIR versus Mg‐H‐SPEEK. f) Volcano plot of DEGs between Mg‐H‐SPEEK and SPEEK. g) Heatmap showing DEGs in Mg‐H‐SPEEK versus SPEEK. h) Enriched pathways by KEGG analysis for Mg‐H‐SPEEK versus SPEEK. i) Schematic diagram of the anti‐inflammatory mechanism of Mg‐H‐SPEEK. j) Enrichment scores for TNF, JAK‐STAT, and NF‐κB pathways in Mg‐H‐SPEEK versus SPEEK. k) Relative expression levels of Cxcl10, Ccl4, and Cxcl2 in Mg‐H‐SPEEK versus SPEEK. ^**^ *p* < 0.01; ^****^ *p* < 0.0001.

To elucidate the mechanisms by which NIR enhances phagocytosis and Mg‐H‐SPEEK exerts anti‐inflammatory effects, we conducted RNA sequencing (RNA‐seq) analysis. Principal Component Analysis (PCA) revealed distinct differences between the Mg‐H‐SPEEK+NIR and Mg‐H‐SPEEK groups, as well as between the Mg‐H‐SPEEK and SPEEK groups (Figure S9, Supporting Information). Specifically, compared to Mg‐H‐SPEEK, the Mg‐H‐SPEEK+NIR group exhibited 254 differentially expressed genes (DEGs), with 76 genes upregulated and 178 genes downregulated (**Figure** **5**a). In contrast, 88 DEGs were identified between Mg‐H‐SPEEK and SPEEK, comprising 20 upregulated and 68 downregulated genes (Figure 5f). A heatmap comparison between Mg‐H‐SPEEK+NIR and Mg‐H‐SPEEK (Figure 5b) highlighted the upregulation of genes related to phagocytosis and inflammation in the Mg‐H‐SPEEK+NIR group. Kyoto Encyclopedia of Genes and Genomes (KEGG) pathway analysis (Figure 5c) revealed significant enrichment of pathways associated with phagocytosis and inflammation, including TNF, FoxO, JAK‐STAT signaling pathway, and Cytokine‐cytokine receptor interaction. These findings suggest that NIR irradiation modulates inflammatory responses and enhances phagocytosis by regulating key signaling pathways. Further RNA‐seq analysis comparing Mg‐H‐SPEEK to SPEEK showed significant downregulation of genes related to inflammation in the Mg‐H‐SPEEK group, as demonstrated by the heatmap (Figure 5g). KEGG pathway analysis (Figure 5h) revealed significant enrichment of pathways such as TNF, JAK‐STAT, NF‐κB, and IL‐17 in Mg‐H‐SPEEK. Gene Set Enrichment Analysis (GSEA) results (Figure 5j) indicated downregulation of these pathways, suggesting that Mg‐H‐SPEEK attenuates inflammation by inhibiting pro‐inflammatory signaling pathways.

To validate these findings, qPCR analysis was conducted to confirm the expression levels of genes associated with phagocytosis and inflammation. Compared to Mg‐H‐SPEEK, the expression of the Traf1 gene was significantly upregulated in Mg‐H‐SPEEK under NIR irradiation (Figure 5e). Traf1 is an important signaling protein that plays a key role in cell survival, apoptosis, and immune regulation. It can mediate the activation of the TNF‐α signaling pathway,^[^ ^28^ ^]^ thereby enhancing the immune effects of M1‐type RAW264.7 macrophages^[^ ^29^ ^]^ and improving their phagocytic capacity. Ccl4, Cxcl2, and Cxcl10 are key pro‐inflammatory chemokines that recruit immune cells such as monocytes, macrophages, neutrophils, and T cells to inflamed sites by binding to G protein‐coupled receptors. Their expression is often upregulated during inflammation and infection, promoting immune cell activation and driving inflammatory responses.^[^ ^30^ ^]^ Our study showed significant downregulation in the expression levels of these pro‐inflammatory genes between Mg‐H‐SPEEK and SPEEK (Figure 5k), demonstrating Mg‐H‐SPEEK's ability to suppress the expression of pro‐inflammatory genes. These results suggest that NIR irradiation enhances phagocytosis, while Mg‐H‐SPEEK suppresses inflammatory responses compared to SPEEK, further corroborating our previous conclusions.

Figure 5d,i presents schematic diagrams illustrating the impact of Mg‐H‐SPEEK on the behavior of RAW264.7 macrophages under NIR irradiation. When NIR is activated, Mg‐H‐SPEEK upregulates the expression of Traf1 in RAW264.7 macrophages via the TNF, FoxO, and JAK‐STAT signaling pathways, as well as Cytokine‐cytokine receptor interactions, enhancing the reprogramming of these macrophages toward the M1 phenotype to effectively eliminate pathogens (Figure 5d). Conversely, when NIR is deactivated, Mg‐H‐SPEEK downregulates the expression of Cxcl10, Ccl4, and Cxcl2 through the TNF, JAK‐STAT, NF‐κB, and IL‐17 pathways, promoting the reprogramming of RAW264.7 macrophages toward the M2 phenotype, thereby supporting tissue healing (Figure 5i).

### 2.7 Evaluation of the In Vivo Antibacterial Effect

**Figure 6:**
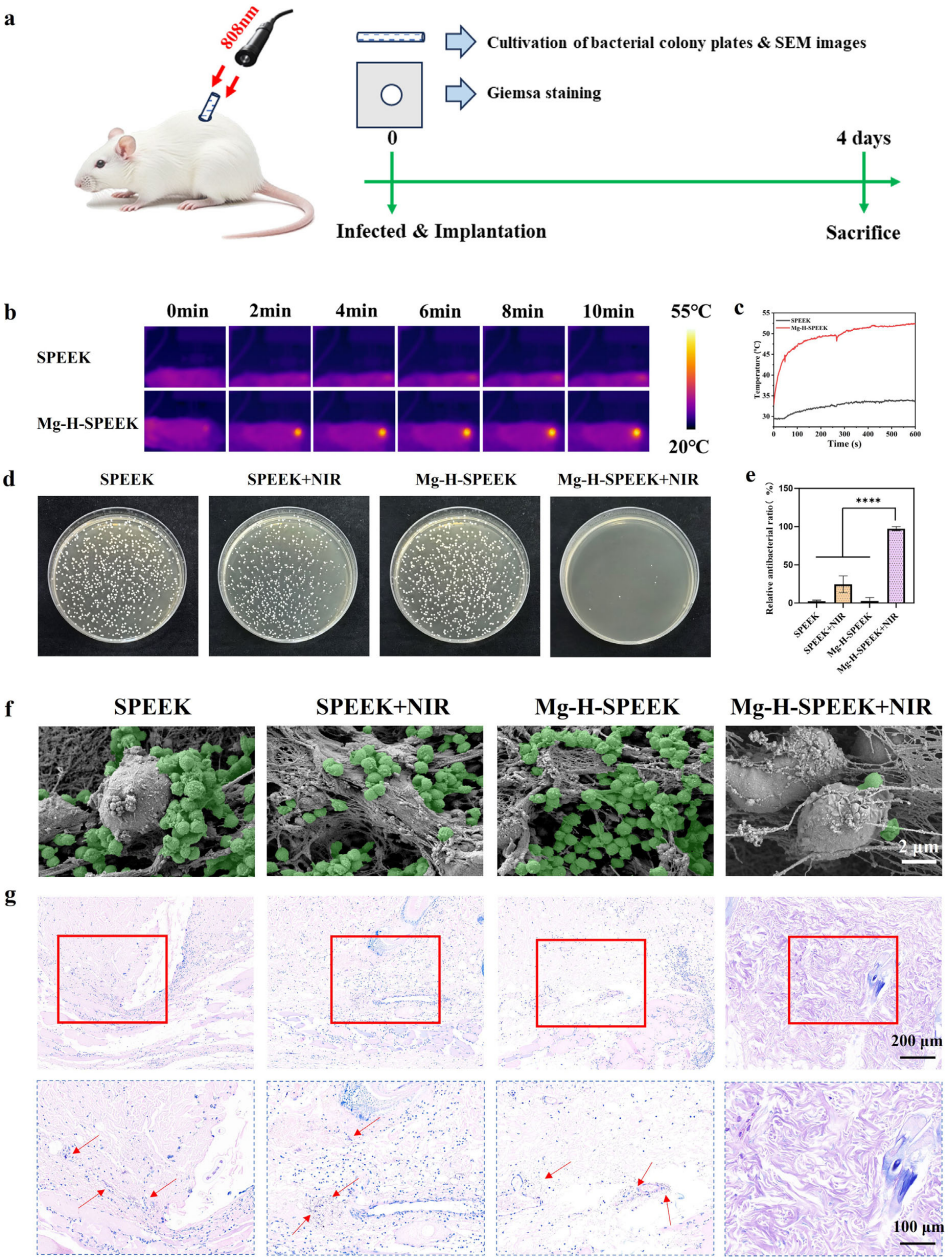
In vivo antibacterial efficacy. a) Schematic diagram illustrating the photothermal antibacterial process of the materials. b) Infrared thermal images showing the temperature distribution at the implantation site in rats. c) Temperature changes at the implantation site in rats under NIR irradiation. d) Photographs of *S. aureus* bacterial colony plates and e) the corresponding relative antibacterial ratios. f) SEM images depicting the surface topography of each material group, with green indicating the presence of observed bacteria. g) Giemsa staining of surrounding tissues after implantation in different groups, the red arrows represent the presence of *S. aureus*. ^****^ *p* < 0.0001.

To comprehensively evaluate the in vivo antibacterial properties of SPEEK, SPEEK+NIR, Mg‐H‐SPEEK, and Mg‐H‐SPEEK+NIR, we established a percutaneous implant infection model (**Figure** **6**a). On the 4 days post‐implantation, we retrieved samples from each group and their surrounding tissues to assess their antibacterial characteristics. Initially, we utilized infrared thermography (Figure 6b) and temperature curve recording (Figure 6c) to quantify the photothermal performance of samples from each group. The results demonstrated that Mg‐H‐SPEEK exhibited significantly superior photothermal properties compared to SPEEK. Specifically, the temperature of Mg‐H‐SPEEK increased by over 10 °C within 100 s under NIR irradiation, whereas the temperature rise of SPEEK did not exceed 5 °C even after 600 s, highlighting the remarkable photothermal characteristics of Mg‐H‐SPEEK. Furthermore, we conducted plate colony counting to evaluate the antibacterial efficacy of Mg‐H‐SPEEK against *S. aureus* under NIR irradiation (Figure 6d). The results indicated that, compared to the SPEEK group, Mg‐H‐SPEEK under NIR irradiation could significantly reduce the number of *S. aureus* colonies, with a relative antibacterial rate exceeding 97%. In contrast, in the absence of NIR irradiation, the difference between Mg‐H‐SPEEK and SPEEK groups was minimal, with a relative antibacterial rate of only 2%. For the SPEEK group, NIR irradiation had a limited impact, with a relative antibacterial rate of 24% (Figure 6e), further emphasizing the superior antibacterial performance of Mg‐H‐SPEEK under NIR irradiation. To further confirm the antibacterial effect of Mg‐H‐SPEEK, we employed SEM to observe bacterial adhesion and growth on the samples from each group (Figure 6f). The SEM images revealed that, compared to the other three groups, the adhesion and growth of bacteria on the Mg‐H‐SPEEK surface were significantly reduced, and the bacterial morphology was incomplete, indicating that the bacterial activity on the Mg‐H‐SPEEK surface was greatly inhibited. Additionally, we performed Giemsa staining to assess the relative number of bacteria in the tissues surrounding the samples from each group, and the results were consistent with the SEM images. A large number of bacteria were found in the tissues surrounding the SPEEK, SPEEK+NIR, and Mg‐H‐SPEEK groups, while the number of bacteria around the Mg‐H‐SPEEK implant under NIR irradiation was extremely low (Figure 6g).

In summary, the results demonstrate that Mg‐H‐SPEEK exhibits potent in vivo antibacterial effects under NIR irradiation.

### 2.8 In Vivo Tissue Healing Assessment in Subcutaneous Implantation Infection Model

**Figure 7:**
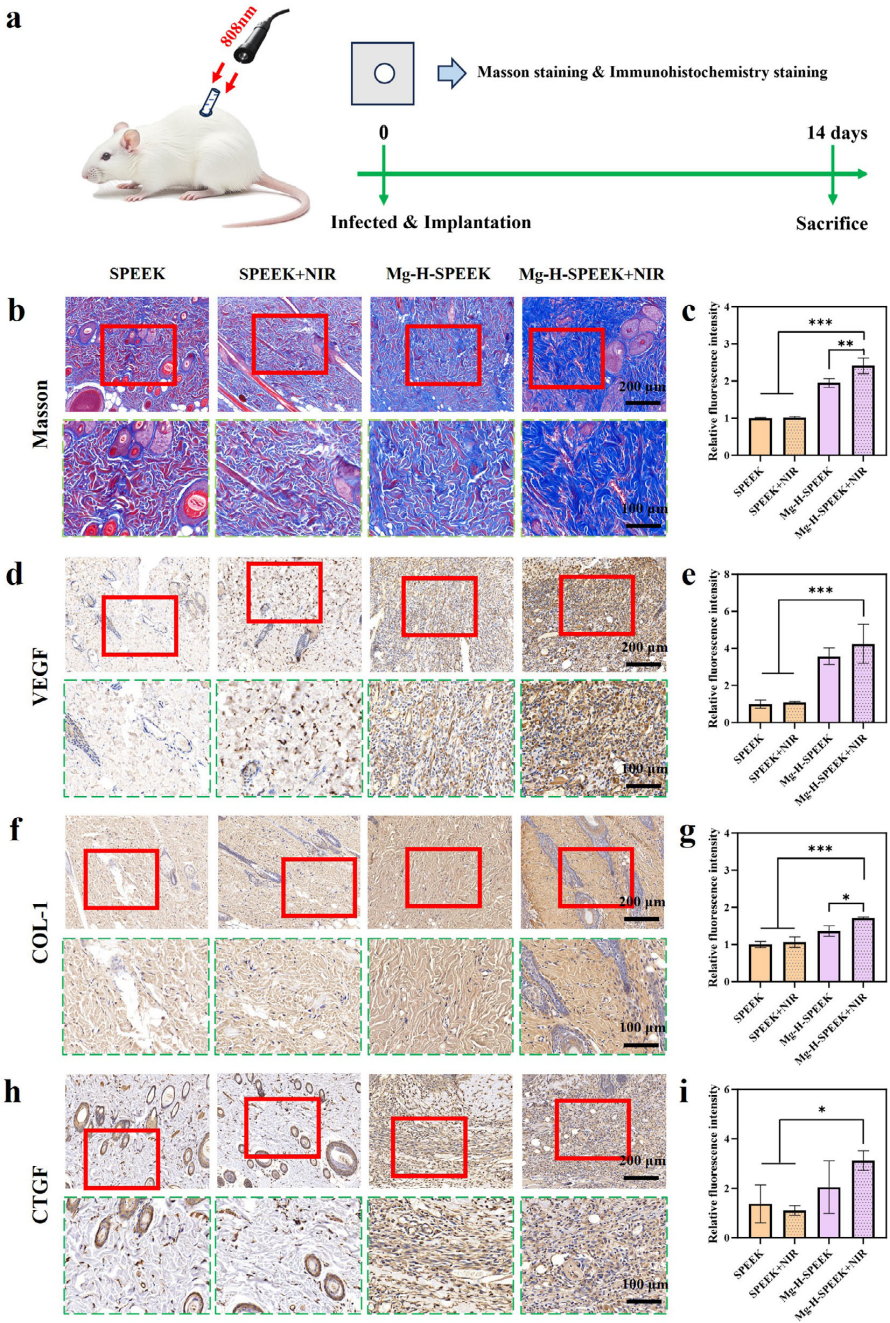
Assessment of tissue healing in vivo under infectious conditions. a) Schematic diagram illustrating the observation of tissue healing in the subcutaneous implantation infection model with NIR irradiation. b) Masson staining images and c) corresponding quantitative analysis. d) Immunohistochemical staining images for VEGF expression and e) corresponding quantitative analysis. f) Immunohistochemical staining images for COL‐1 expression and g) corresponding quantitative analysis. h) Immunohistochemical staining images for CTGF expression and i) corresponding quantitative analysis. ^*^ *p* < 0.05; ^**^ *p* < 0.01; ^***^ *p* < 0.001.

To comprehensively evaluate the in vivo tissue healing capabilities of SPEEK, SPEEK+NIR, Mg‐H‐SPEEK, and Mg‐H‐SPEEK+NIR, we established a subcutaneous implantation infection model. As depicted in **Figure** **7**a, 14 days post‐implantation, we harvested samples from the surrounding tissues of each group to assess their healing properties. Using Masson staining (Figure 7b) and quantitative analysis (Figure 7c), we quantified collagen deposition and tissue organization across each group. The findings demonstrated that Mg‐H‐SPEEK significantly outperformed SPEEK in terms of collagen deposition. Specifically, both Mg‐H‐SPEEK and Mg‐H‐SPEEK+NIR groups exhibited enhanced collagen formation, with NIR irradiation further promoting collagen synthesis in the Mg‐H‐SPEEK+NIR group. This highlights the remarkable antibacterial and healing properties of Mg‐H‐SPEEK under NIR irradiation. In contrast, the SPEEK group showed minimal collagen deposition regardless of NIR treatment, underscoring its lack of photothermal effect and its inability to effectively facilitate soft tissue healing.

Furthermore, we conducted immunohistochemical staining for VEGF (vascular endothelial growth factor) (Figure 7d) and quantitative analysis (Figure 7e) to assess angiogenesis in the samples from each group. VEGF, a pivotal factor in angiogenesis, is crucial for tissue healing and regeneration. The results indicated that Mg‐H‐SPEEK significantly enhanced VEGF expression compared to the SPEEK group, with NIR irradiation further increasing VEGF expression in the Mg‐H‐SPEEK group. For the SPEEK group, NIR irradiation had a limited impact on angiogenesis, with only a slight increase observed, thereby reinforcing the superior photothermal antibacterial performance of Mg‐H‐SPEEK.

To further substantiate the tissue healing effects of Mg‐H‐SPEEK, we employed immunohistochemical staining to examine the expression of COL‐1 (Figure 7f,g) and CTGF (Figure 7h,i) in samples from each group. COL‐1 (Type I Collagen), a major component of the extracellular matrix, is essential for tissue structural stability and strength, while CTGF (Connective Tissue Growth Factor) plays a significant role in regulating the deposition and remodelling of the extracellular matrix. The staining images revealed that, compared to SPEEK, the expression of COL‐1 and CTGF on the surface of Mg‐H‐SPEEK was significantly increased, with NIR irradiation further enhancing the expression of COL‐1 and CTGF in Mg‐H‐SPEEK. In contrast, NIR irradiation had no significant effect on SPEEK. This suggests that the activity of fibroblasts and the deposition of the extracellular matrix on the surface of Mg‐H‐SPEEK were significantly augmented.

In summary, the results indicate that Mg‐H‐SPEEK enhances collagen synthesis, angiogenesis, and overall tissue healing, particularly under NIR irradiation. This is attributed to its excellent photothermal antibacterial properties.

### 2.9 In Vivo Immunological Responses and Tissue Healing Assessment in Subcutaneous Implantation Model

**Figure 8:**
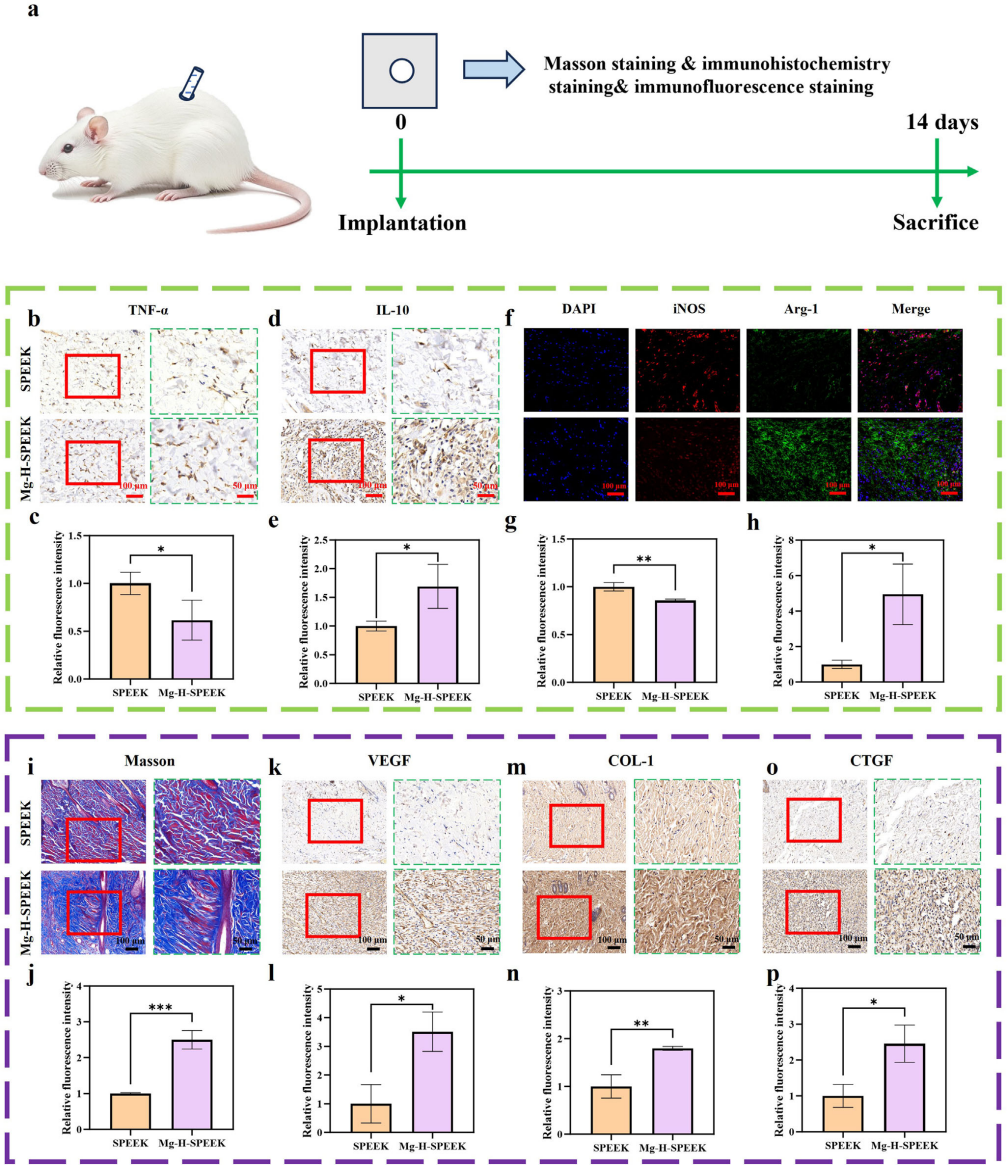
Assessment of immunological responses and tissue healing in vivo. a) Schematic diagram illustrating the observation of immunological responses and tissue healing in the subcutaneous implantation model. b) Immunohistochemical staining images for TNF‐α expression and c) corresponding quantitative analysis. d) Immunohistochemical staining images for IL‐10 expression and e) corresponding quantitative analysis. f) Immunofluorescence staining images for iNOS/Arg‐1 expression and g,h) corresponding quantitative analysis. i) Masson staining images depicting collagen fiber arrangement and j) corresponding quantitative analysis. k) Immunohistochemical staining images for VEGF expression and l) corresponding quantitative analysis. m) Immunohistochemical staining images for COL‐1 expression and n) corresponding quantitative analysis. o) Immunohistochemical staining images for CTGF expression and p) corresponding quantitative analysis. ^*^ *p* < 0.05; ^**^ *p* < 0.01; ^***^ *p* < 0.001.

To delve into the immunological responses and tissue healing effects of SPEEK and Mg‐H‐SPEEK in vivo, we established a subcutaneous implantation model. As depicted in **Figure** **8**a, after 14 days post‐implantation, we harvested surrounding tissue samples from each group to evaluate their immunological responses and healing properties.

We initially assessed the immunological responses using immunohistochemistry and immunofluorescence techniques. The immunohistochemical results for TNF‐α and IL‐10 indicated that the expression of TNF‐α was significantly lower in the Mg‐H‐SPEEK group compared to the SPEEK group (Figure 8b,c). In contrast, IL‐10 showed increased expression in the Mg‐H‐SPEEK group (Figure 8d,e), suggesting that Mg‐H‐SPEEK facilitates a shift from a pro‐inflammatory to an anti‐inflammatory state. Furthermore, the expression levels of iNOS and Arg‐1 (Figure 8f), as detected by immunofluorescence, revealed a decrease in iNOS and an increase in Arg‐1 in the Mg‐H‐SPEEK group (Figure 8g,h), indicating that Mg‐H‐SPEEK promotes the macrophage reprogramming toward the M2 phenotype, which is more conducive to inflammation resolution and tissue healing.

Subsequently, we evaluated tissue healing through Masson staining and immunohistochemical staining. Masson staining (Figure 8i) and subsequent quantitative analysis (Figure 8j) unveiled enhanced collagen fiber arrangement in the Mg‐H‐SPEEK group, confirming increased collagen deposition and tissue formation, which is crucial for the stability and strength of tissue structure. The expression of VEGF (Figure 8k) and its quantitative analysis (Figure 8l) demonstrated elevated vascular endothelial growth factor levels in the Mg‐H‐SPEEK group, suggesting its potential to stimulate angiogenesis, thereby providing better nutritional and oxygen supply to the tissues. The expression of COL‐1 (Figure 8m) and its quantitative analysis (Figure 8n) also increased in the Mg‐H‐SPEEK group, indicating enhanced fibrogenesis, which is essential for tissue healing. The expression of CTGF (Figure 8o) and its quantitative analysis (Figure 8p) were significantly upregulated in the Mg‐H‐SPEEK group, further confirming the role of Mg‐H‐SPEEK in promoting tissue healing by fostering fibroblast activity and extracellular matrix deposition.

Additionally, histological analysis in Figure S10 (Supporting Information) of key organs such as the heart, liver, spleen, lungs, and kidneys reveals normal morphology without signs of inflammation, injury, or necrosis, indicating excellent biocompatibility of the implants. This comprehensive assessment highlights the safety and efficacy of the Mg‐H‐SPEEK implant.

In summary, these findings lead us to conclude that Mg‐H‐SPEEK significantly enhances immunomodulation and tissue healing by modulating inflammatory factors, promoting macrophage reprogramming, improving collagen synthesis, stimulating angiogenesis, and facilitating overall tissue healing.

## 3 Conclusion

This study successfully developed Mg‐H‐SPEEK, a novel PEEK derivative that integrates sulfonation with a two‐step plasma immersion ion implantation process. This innovative approach creates a porous, graphite‐like film embedded with MgO. Comprehensive in vitro and in vivo experiments have demonstrated its dynamic regulatory capabilities. Under NIR irradiation, the “photon trap” characteristic of Mg‐H‐SPEEK enhances the photothermal conversion ability of the graphite‐like film. Local high temperature exhibits significant photothermal antibacterial effects and enhances macrophage phagocytosis by promoting reprogramming toward the M1 phenotype. Conversely, in the absence of NIR stimulation, it facilitates macrophage reprogramming toward the M2 phenotype, thereby supporting tissue healing. RNA‐seq analysis further elucidated the mechanisms underlying its enhanced phagocytosis following NIR irradiation and its immune regulatory effects in the absence of NIR. These findings highlight that Mg‐H‐SPEEK can rapidly eliminate pathogens during the initial infection phase and promote tissue healing during the healing phase, depending on whether NIR stimulation is present or not.

## 4 Experimental Section

### Sample Preparation

Medical‐grade PEEK materials were sourced from Jiangsu Junhua Company. The samples were fabricated into various configurations: disc‐shaped samples with dimensions of φ12 mm × 1 mm were utilized for in vitro experiments, whereas rod‐shaped samples measuring φ2 mm × 10 mm were employed for in vivo animal studies. Each sample underwent a meticulous cleaning process, involving sequential 10‐min immersions in acetone, ethanol, and ultrapure water to ensure surface purity. To introduce a 3D porous structure on the PEEK surface, sulfonation was performed using concentrated sulfuric acid, yielding samples designated as SPEEK. Subsequently, hydrogen ion implantation was conducted on the SPEEK surface to produce H‐SPEEK. The samples were placed in a PIII vacuum chamber (PBII‐2 Multifunctional Composite Surface Modification System, Zhonghe Tongchuang Technology Co., Ltd., China), where hydrogen plasma was implanted into PEEK according to the core PIII parameters detailed in Table S1 (Supporting Information). Surface modification was further extended to Mg‐SPEEK and Mg‐H‐SPEEK samples via magnesium plasma immersion ion implantation on PEEK and H‐SPEEK surfaces, respectively. These samples were processed in a PIII vacuum chamber (PBII‐2 Multifunctional Composite Surface Modification System, China National Nuclear Innovation Technology Co., Ltd., China). Hydrogen plasma was initially injected into PEEK based on the core PIII parameters listed in Table S2 (Supporting Information) to obtain the desired samples. To explore the photon trapping characteristics of the materials, H‐PIII treatment was initially applied to both PEEK and SPEEK. The injection voltage was set at 40 kV, with the remaining parameters specified in Table S1 (Supporting Information). The treated samples were labeled as H‐P and H‐SP, respectively. Additionally, the sulfonation time of PEEK was varied to 4, 8, and 12 min to create samples with different sulfonation durations. These samples were then subjected to hydrogen ion injection treatment using the parameters outlined in Table S1 (Supporting Information) (with an injection voltage of 40 kV), resulting in samples named H‐SP‐4, H‐SP‐8, and H‐SP‐12.

### Surface Morphology and Elemental Composition Analysis

The surface morphology and elemental composition of the samples were examined using a Field Emission Scanning Electron Microscope (FE‐SEM; SU‐8200, Hitachi, Japan). Raman scattering spectra were collected using a HORIBA Jobin Yvon HR800 Raman spectrometer (France) with a 514 nm excitation laser to assess the quality of the treated and untreated PEEK samples. The surface chemical composition was analyzed using a Fourier Transform Infrared Spectrometer (FT‐IR; FTIR‐7600, Lambda Scientific, Australia). The surface chemical states were investigated using X‐ray Photoelectron Spectroscopy (XPS) on an upgraded RBD PHI‐5000C ESCA system (Perkin Elmer) with Mg Kα radiation (h*ν* = 1253.6 eV). The surface wettability of the samples was measured using a sessile drop of distilled water with an automatic contact angle meter (model SL200B, Solon Information Technology Co., Ltd., China). The crystalline phases of the samples were characterized by X‐ray Diffraction (XRD;D/Max,Rigaku, Japan). Electrical measurements were conducted using a semiconductor parameter analyzer (Agilent B1500A, USA) under dark room temperature conditions. Optical measurements were performed using a UV–vis–NIR spectrophotometer (Lambda 750, USA). The photothermal properties were evaluated using a laser with a power density of 0.8 W cm^−^^2^ and a wavelength of 808 nm (MDL‐H) in combination with an infrared thermal imager (Fotric 285s, USA) to record the temperature changes, infrared thermograms, and photothermal cycling curves in both air and 1 mL water environments.

### Bacterial Culture

To evaluate the antibacterial properties of the samples, Gram‐positive Staphylococcus aureus (*S. aureus*, ATCC 25 923) and Gram‐negative Escherichia coli (*E. coli*, ATCC 25922) were employed. *S. aureus* was cultured in Tryptic Soy Broth, while *E. coli* was cultured in Luria‐Bertani medium. The samples were sterilized using 75%alcohol for 2 h and then dried.

### Plate Colony Counting

The materials were inoculated with bacteria at a concentration of 1 × 10⁷ CFU mL^−1^ and incubated for 24 h. Following this, the samples were washed with saline to eliminate any planktonic bacteria. Subsequently, half of the samples were exposed to near‐infrared (NIR)irradiation at an intensity of 0.8 W cm^−^^2^ for 10 min, while the remaining samples were left unirradiated and served as controls. After irradiation, the bacteria were carefully separated from the samples. A 100 µL aliquot of the diluted bacterial suspension was then spread onto agar plates and incubated at 37 °C for 18 h. The agar plates were photographed, and the number of bacterial colonies was counted to assess the antibacterial efficacy.

### Bacterial Morphology Observation

For microscopic examination, bacterial samples were prepared for both scanning electron microscopy (SEM) and transmission electron microscopy (TEM). Initially, the samples underwent NIR treatment in physiological saline for 5 min, followed by rinsing. Subsequently, they were fixed with 2.5% glutaraldehyde solution at 4 °C for 5 min. Dehydration was achieved using a gradient ethanol series (30%–100% V/V), with each step lasting for 10 min. For SEM analysis, the dehydrated samples were subjected to critical point drying to remove residual solvents. They were then mounted on aluminum stubs, coated with a thin layer of gold using a sputter coater, and examined under a scanning electron microscope (model S3400, HITACHI, Japan). For TEM analysis, the dehydrated samples were embedded in epoxy resin (Spurr's resin). Ultrathin sections, ≈70 nm in thickness, were prepared using an ultramicrotome (Leica EM UC7, Leica Microsystems, Germany) and placed on copper grids. Contrast enhancement was achieved by staining the sections with uranyl acetate and lead citrate. TEM imaging was conducted using a transmission electron microscope (Hitachi H‐7650, HITACHI, Japan) operated at 80 kV. The resulting images were captured using a CCD camera (Gatan Orius SC1000, Gatan Inc., USA) attached to the microscope.

### Cell Culture

RAW264.7 macrophages (ATCC Cat# TIB‐71, RRID: CVCL_0493) and L929 fibroblasts (NCTC clone 929, RRID: CVCL_0462) were cultivated in T‐25 flasks using complete culture medium, which consisted of 85%high‐glucose DMEM, 10%FBS‐Premium (FBS‐UP500), NEWZERUM Ltd), and 1%penicillin‐streptomycin (Gibco, USA). The cells were maintained in a humidified incubator with 5%CO_2_ at 37 °C. The culture medium was replaced every 3 days, and the cells were passaged at a 1:2 split ratio.

### Cell Proliferation

Cell proliferation was evaluated using the alamarBlue assay (AbD Serotec Ltd., UK). Cell suspensions (1 × 10⁵ cells mL^−1^,1 mL) were seeded onto various specimen surfaces and cultured for 3 days. At the time point, the specimens were rinsed twice with PBS, followed by the addition of 0.5 mL of medium containing 10%alamarBlue for 2 h. The fluorescence intensity was measured at an excitation wavelength of 560 nm and an emission wavelength of 590 nm.

### Phagocytosis Assay

RAW264.7 macrophages were seeded onto SPEEK and Mg‐H‐SPEEK samples at a density of 1 × 10⁵ cells per specimen and cultured for 3 days. Cells were subjected to NIR irradiation (0.8 W cm^−^^2^) for 5 min in complete medium, followed by incubation at 37 °C for 6 h. Nonirradiated cells were used as controls. Subsequently, 5 µg of green fluorescent polystyrene microspheres (PS,100 nm) were added to each well, gently mixed, and co‐cultured for 3 h. Cells were then rinsed with PBS to remove extracellular PS, treated with 0.05%trypsin for 3 min, and resuspended in complete medium. After washing with PBS, nuclei were stained with DAPI (10 min, dark, room temperature) and fixed in 4%paraformaldehyde. A 50 µL cell suspension was dropped onto slides, and phagocytic efficiency was assessed by counting the number of PS microspheres per nucleus using a Leica LSM 800 confocal microscope (Leica, Germany).

### Cytokine Secretion Assays

RAW264.7 macrophages and L929 fibroblasts were seeded onto the surfaces of various samples at a density of 1 × 10⁵ cells per specimen and cultured for 3 days. Subsequently, the samples were divided into different groups and stabilized in an incubator for 6 h. The supernatants were then collected in 1.5 mL EP tubes. Enzyme‐linked immunosorbent assay (ELISA; Anogen) was employed to quantify the levels of cytokines, including IL‐4, IL‐10, IL‐18, and TNF‐𝛼 in RAW264.7 macrophages, and TGF‐𝛽 in L929 fibroblasts.

### Co‐Culture of RAW264.7 Macrophages and L929 Fibroblasts

RAW264.7 macrophages and L929 fibroblasts were co‐cultured in 24‐well plates. RAW264.7 cells were seeded in the upper chamber at a density of 7 × 10^3^ cells per specimen in 1 mL of complete DMEM medium and allowed to adhere for 24 h. Subsequently, L929 fibroblasts were introduced into the lower chamber at a density of 1 × 10⁴ cells per specimen in 1 mL of complete DMEM medium. The co‐culture was maintained at 37 °C in a 5%CO_2_ humidified atmosphere for 7 days, with medium changes every 2–3 days. After 3 days, the RAW264.7 macrophages were removed from the upper chamber.

### Real‐Time Polymerase Chain Reaction Analysis

The expression of inflammation‐related genes in RAW264.7 macrophages and soft tissue‐promoting genes in L929 fibroblasts on the surfaces of samples was analyzed by real‐time polymerase chain reaction (RT‐PCR). RAW264.7 macrophages were cultured on the samples at a density of 1 × 10⁵ cells per specimen for 3 days, and L929 fibroblasts were cultured at the same density for 7 days. Total RNA was extracted using the EZ‐press RNA Purification Kit (EZBioscience, USA) and reverse transcribed to cDNA using the Color Reverse Transcription Kit (EZBioscience, USA). Quantitative RT‐PCR was performed using 2 × Color SYBR Green qPCR Master Mix (EZBioscience, USA). Target gene expression was normalized to the housekeeping gene GAPDH and analyzed using the 2^−^ΔΔCt method.

### Collagen Secretion

The collagen secretion on various sample surfaces was assessed using Sirius Red staining. L929 fibroblasts were seeded at a density of 1 × 10⁴ cells per specimen and cultured for 7 days. The cells were subsequently rinsed with PBS, fixed in 4%paraformaldehyde, stained with 0.1%Sirius Red, and washed with 0.1 m acetic acid. For quantitative analysis, the stain was extracted using a mixture of NaOH and methanol, and the optical density was measured at 492 nm.

### RNA‐Sequencing

For the investigation of differential gene expression between Mg‐H‐SPEEK versus SPEEK and Mg‐H‐SPEEK+NIR versus Mg‐H‐SPEEK, transcriptomic analysis was performed. Total RNA was isolated from the samples utilizing the Trizol reagent (Invitrogen) following the manufacturer's guidelines. Subsequently, the RNA samples were subjected to high‐throughput sequencing and subsequent bioinformatic analysis by OE Biotech (Shanghai, China). The criteria for significance in gene expression changes were set at a fold change exceeding 1.5 and a p‐value below 0.05. All data processing steps were executed on the OE Biotech cloud‐based platform, accessible at https://cloud.oebiotech.cn/task/.

### Percutaneous Implantation Infected Model

The experimental protocols have been approved by the Experimental Animal Management Committee of Shanghai Chengxi Biotechnology Co., Ltd. The ethical approval number was CX052403055. Eight‐week‐old male SD rats were used to create percutaneous implantation infected models. SPEEK and Mg‐H‐SPEEK cylinders were immersed in a Staphylococcus aureus suspension (5 × 10⁶ CFU mL^−1^) and co‐incubated for 1 h. The rats were randomly divided into two groups and anesthetized with 10% sodium pentobarbital (35 mg kg^−1^). After depilation and disinfection with iodophor, a Kirschner wire (Φ2 mm) was used to create a skin wound for cylinder insertion, which was then secured with photocurable adhesive. The groups were further divided into NIR‐ and NIR+ subgroups based on whether they received 5 min of NIR irradiation post‐surgery. An infrared thermal imager monitored temperature changes and captured thermal images of the implants during the procedure. After surgery, the rats were housed in a clean environment for a specified recovery period.

On day 4 post‐implantation, samples and their surrounding tissues were retrieved from each group. The samples were separated from the surrounding soft tissues. The separated soft tissues were used for Giemsa staining, while the implanted samples were collected for plate colony counting and scanning electron microscopy (SEM) analysis to assess antimicrobial properties. On day 14, tissues were collected for Masson staining and immunohistochemical analysis. Additionally, histological evaluation was performed on the heart, liver, kidneys, lungs, and spleen to assess the biocompatibility of the materials in each group.

### Percutaneous Implantation Model

Eight‐week‐old male SD rats were used for percutaneous implantation models. SPEEK and Mg‐H‐SPEEK cylinders were sterilized with 75% alcohol for 2 h, then dried. Rats were divided into two groups, anesthetized with 10% sodium pentobarbital (35 mg kg^−1^), and underwent depilation and iodophor disinfection. A Kirschner wire (Φ2 mm) created a skin wound for cylinder insertion, secured with photocurable adhesive. Groups were divided into NIR‐ and NIR+ based on 5‐min NIR irradiation post‐surgery.

On day 14, tissues were collected for Masson staining, immunohistochemical analysis, and immunofluorescence.

### Statistical Analysis

Data were analyzed using GraphPad Prism10. Results were expressed as mean ± SD. One‐way ANOVA was used to assess significant differences. Significance levels: *p* < 0.05 (^*^), *p* < 0.01 (^**^), *p* < 0.001 (^***^), and *p* < 0.0001 (^****^).

## Conflict of Interest

The authors declare no conflict of interest.

## Supporting information



Supporting Information

## Data Availability

Research data are not shared.
