# Supplementary material for: Near‐Infrared Switch‐Driven Macrophage Dynamically Reprogramming for Anti‐Infection and Tissue Healing on Polyetheretherketone Implants
Source: Adv Sci (Weinh). 2025 Nov 4;12(47):e12592. doi: 10.1002/advs.202512592 (PMC12713068; doi:10.1002/advs.202512592)
Supplement: Supplementary file 1 — Supporting Information [file ADVS-12-e12592-s001.docx]

**Supporting Information**

**Near-Infrared Switch-Driven Macrophage Dynamically Reprogramming for Anti-infection and Tissue Healing on polyetheretherketone Implants**

Yinghao Wu^a,1^, Shuaiqi Jiang^b, c, 1^, Jibing He^a,1^, Ji Tan^b,^*, Junjie Zhou^b, c^ , Zhipeng Zou^a^, Jiaxing Wangª, Jia Jiangª, Xuanyong Liu^b,d,^*, and Xiaochun Peng^a,^*

^a^Shanghai Sixth People's Hospital Affiliated to Shanghai Jiao Tong University School of Medicine, Shanghai 200233, China.

^b^State Key Laboratory of Advanced Ceramics, Shanghai Institute of Ceramics, Chinese Academy of Sciences, Shanghai 200050, China.

^c^Center of Materials Science and Optoelectronics Engineering, University of Chinese Academy of Sciences, Beijing 100049, China.

^d^School of Chemistry and Materials Science, Hangzhou Institute for Advanced Study, University of Chinese Academy of Sciences, Hangzhou 310024, China.

^1^These authors contributed equally to this work.

*Corresponding authors:

Xiaochun Peng, dr.xcpeng@shsmu.edu.cn; Xuanyong Liu, xyliu@mail.sic.ac.cn; Ji Tan, tanji@mail.sic.ac.cn

Figure S1a presents the Mg²⁺ release profiles across a spectrum of pH levels (6.5, 7.4, and 8.5), and Figure S4b shows the Mg²⁺ release under various NIR irradiation durations (0, 3, 5, and 7 minutes). Collectively, these graphs indicate that Mg²⁺ ion release is fairly stable and is not significantly influenced by changes in pH or the temperature conditions resulting from NIR irradiation, implying a robust Mg²⁺ release kinetics that is largely independent of these variables.


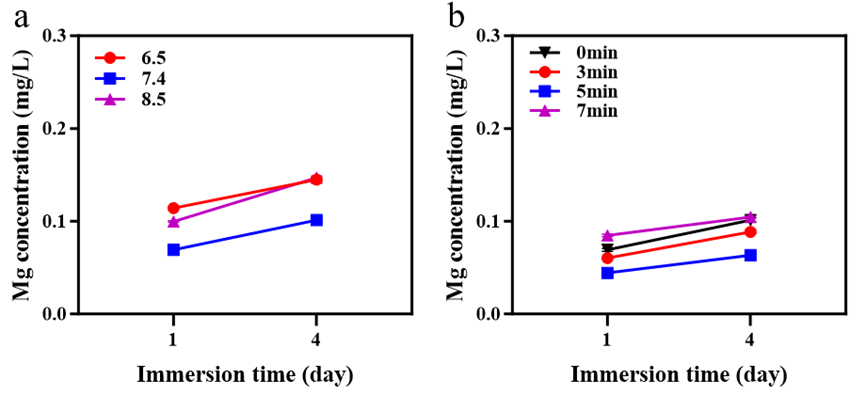


**Figure S1.** The release profile of Mg²⁺ under varying pH and temperature conditions. (a) Cumulative release results of Mg²⁺ from Mg-H-SPEEK at different pH. (b) Cumulative release results of Mg²⁺ released from Mg-H-SPEEK under different near-infrared irradiation times.

The fourier Transform Infrared Spectroscopy (FTIR) spectra represent the following samples: SPEEK (black line), H-SPEEK (red line), Mg-SPEEK (blue line), and Mg-H-SPEEK (purple line). The dashed box highlights a region of interest where significant differences in the chemical composition or functional groups among the samples can be observed. After PIII treatment, carbonyl characteristic peaks (C=O) appeared at 1710 cm^-1^ in H-SPEEK, Mg-SPEEK, and Mg-H-SPEEK, indicating a possible change in their surface structure.


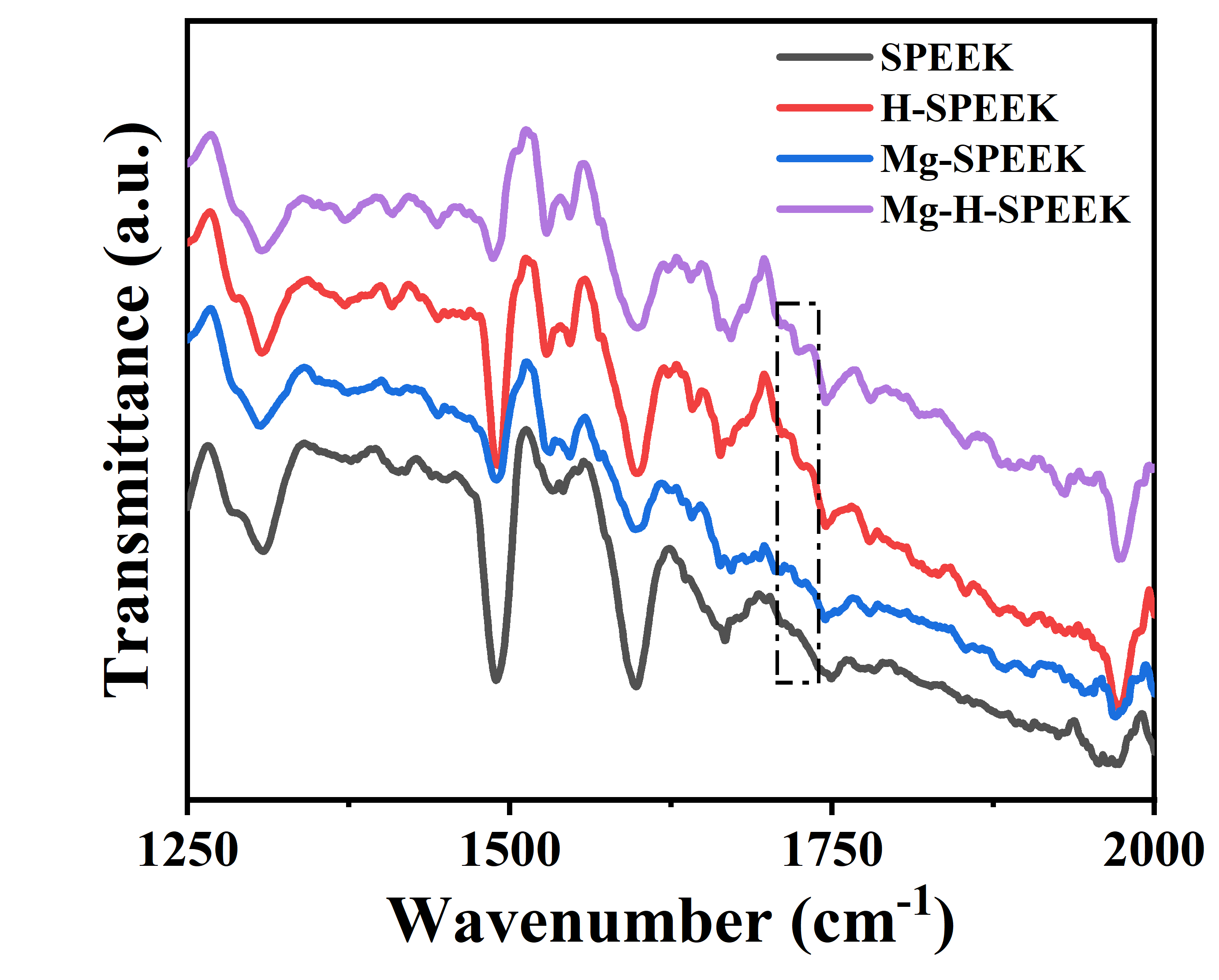


**Figure S2.** FTIR spectra of SPEEK, H-SPEEK, Mg-SPEEK, and Mg-H-SPEEK in the wavenumber range of 1250 to 2000 cm⁻¹.

As shown in Figure S3a, compared with SP, the fundamental morphology of the H-SP is preserved after PIII treatment. Compared with H-P, H-SP displays a three-dimensional porous structure. Figure S3b and Figure S3c demonstrate that H-SP, with its porous structure acting as an effective light-trapping scaffold, exhibits superior light absorption and photothermal performance among PEEK, SPEEK, H-P, and H-SP.


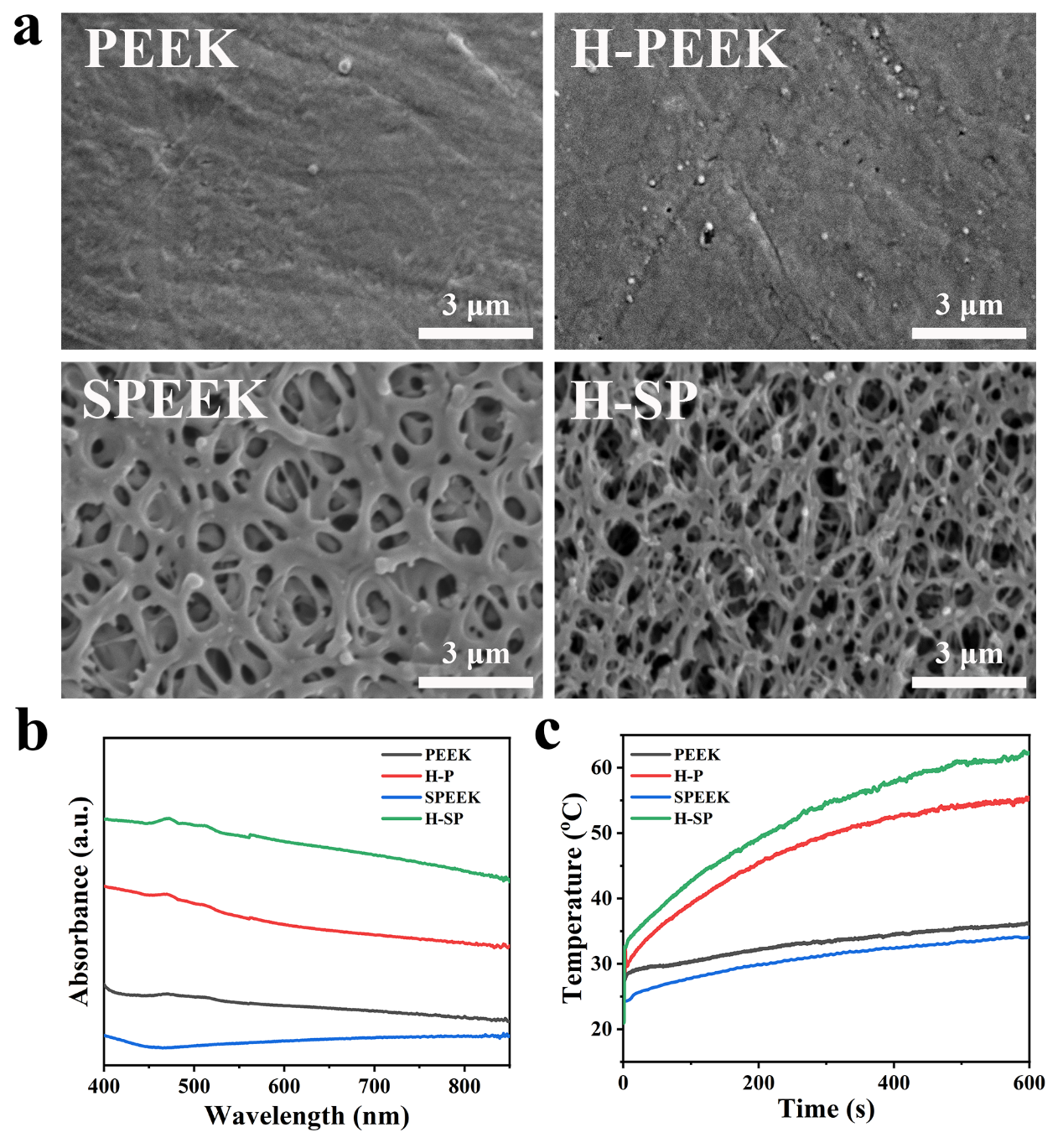


**Figure S3.** Characterization of PEEK and its modified versions. (a) SEM images; (b) UV-vis spectra and (c) temperature-time profiles under NIR for PEEK, SPEEK, H-P and H-SP.

By adjusting the sulfonation time, we have created a range of PEEK surfaces with varying pore structures, allowing us to investigate the relationship between pore structure and light absorption.

As shown in Figure S4a, the porosity and pore size of the material surface increase with the extended sulfonation time. Figure S4b and Figure S4c further demonstrate the light absorption and photothermal conversion capabilities of the samples. Among them, H-SP-12 exhibits the most favorable performance. This is consistent with the general principle that higher porosity and larger specific surface area facilitate more efficient light absorption.


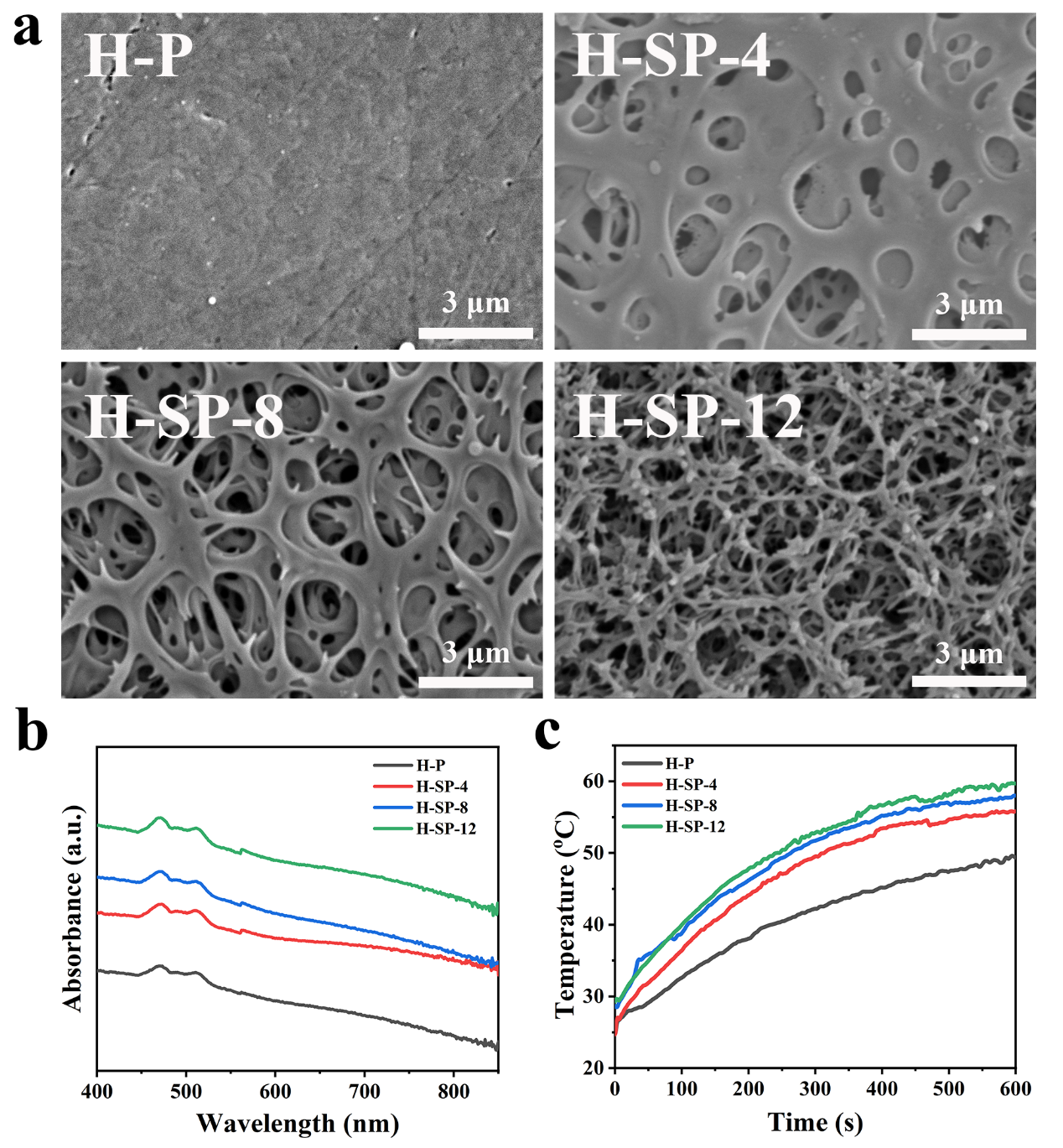


**Figure S4.** Characterization of H-PEEK and its sulfonated variants with different sulfonation times. (a) SEM images; (b) UV-vis spectra and (c) temperature-time profiles under NIR for H-P, H-SP-4, H-SP-8 and H-SP-12.

Both RAW264.7 macrophages and L929 fibroblasts exhibited significantly enhanced cell proliferation on H-SPEEK, Mg-SPEEK, and Mg-H-SPEEK compared to SPEEK (Figure S5 and Figure S6), highlighting their superior cytocompatibility.

**Figure S5.** The fluorescent density of alamarBlue for RAW264.7 macrophages cultured for 3 days. (*was p < 0.05)

**Figure S6.** The fluorescent density of alamarBlue for L929 fibroblasts cultured for 3 days in Single-Culture and Co-Culture systems. (*** was p < 0.001)

Sirius Red staining showed that in Single-Culture, L929 fibroblasts secreted more collagen on Mg-H-SPEEK and H-SPEEK. In Co-Culture, Mg-H-SPEEK and Mg-SPEEK exhibited higher collagen expression than the other groups (Figure S7), indicating their enhanced M2 macrophage reprogramming and subsequent collagen secretion.

**Figure S7.** Quantitative analysis of collagen in L929 fibroblasts using Sirius Red Staining (N=5, **** was p < 0.0001)

The scratch assay (Figure S8) on L929 fibroblasts further demonstrated that, whether in Single-Culture or Co-Culture systems, Mg-H-SPEEK exhibited the most effective wound-healing performance.

**
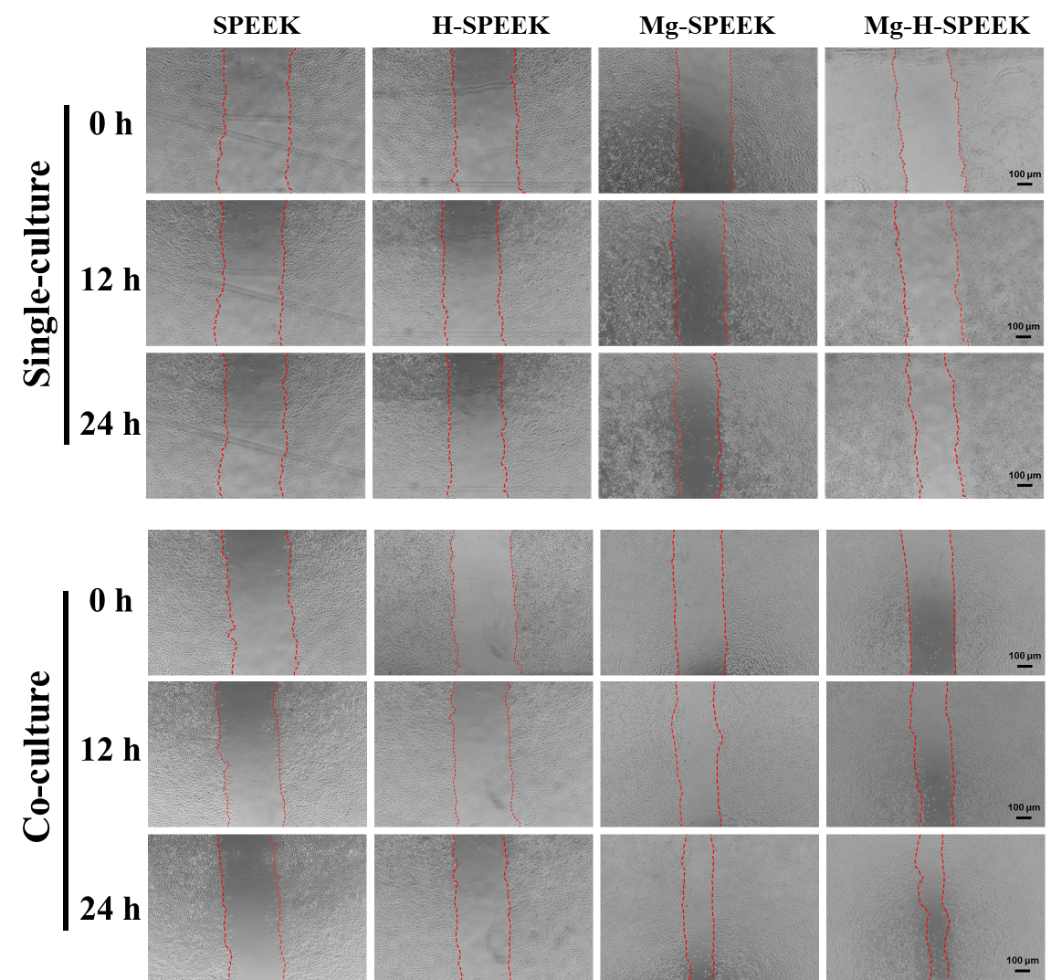
**

**Figure S8.** The scratch assay for L929 fibroblasts migration analysis.

Principal Component Analysis (PCA) revealed distinct differences between the Mg-H-SPEEK + NIR and Mg-H-SPEEK groups, as well as between the Mg-H-SPEEK and SPEEK groups (Figure S9). The first principal component (PC1) accounted for the majority of the variance, highlighting the significant separation between these groups.


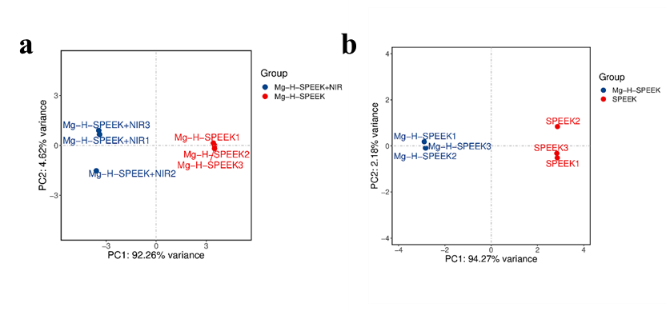


**Figure S9.** (a) PCA of Mg–H–SPEEK+NIR and Mg–H–SPEEK groups. (b) PCA of Mg–H–SPEEK and SPEEK groups.


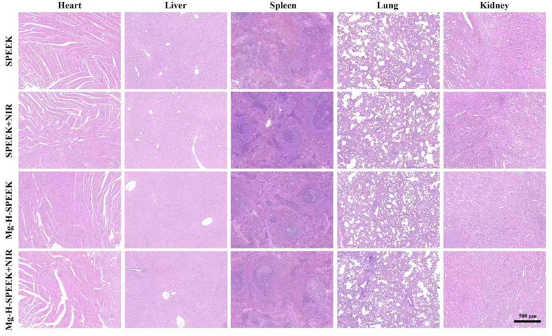
The histological analysis in Figure S10 of key organs such as the heart, liver, spleen, lungs, and kidneys reveal normal morphology without signs of inflammation, injury, or necrosis, indicating excellent biocompatibility of the implants.

**Figure S10.** H&E images from major organs (heart, liver, spleen, lung, and kidney) of the implants.

**Table S1.** Process parameters for H-PIII

| **Implantation parameter** | **Value** |
| --- | --- |
| Voltage | -30 kV |
| Frequency | 100 Hz |
| Pulse width | 30 μs |
| Time | 60 min |
| Background vacuum | 5 × 10⁻³ Pa |

**Table S2.** Process parameters for Mg-PIII

| **Implantation parameter** | **Value** |
| --- | --- |
| Cathode Pulse Width | 500 μs |
| Voltage | -15 kV |
| Frequency | 8 Hz |
| Pulse width | 500 μs |
| Time | 60 min |
| Background vacuum | 5 × 10⁻³ Pa |

**Table S3.** Primer sequence

| **Gene** | | **Primer** | **Sequences (5’-3’)** |
| --- | --- | --- | --- |
| GAPDH | | Forward  Reverse | AGGTCGGTGTGAACGGATTTG  GGGGTCGTTGATGGCAACA |
| CD206 | | Forward  Reverse | CTCTGTTCAGCTATTGGACGC  TGGCACTCCCAAACATAATTTGA |
| IL-6 | | Forward  Reverse | CTGCAAGAGACTTCCATCCAG  AGTGGTATAGACAGGTCTGTTGG |
| IL-4 | | Forward  Reverse | GGTCTCAACCCCCAGCTAGT  GCCGATGATCTCTCTCAAGTGAT |
| TLR4 | | Forward  Reverse | ATGGCATGGCTTACACCACC  GAGGCCAATTTTGTCTCCACA |
| MyD88 | | Forward  Reverse | TCATGTTCTCCATACCCTTGGT  AAACTGCGAGTGGGGTCAG |
| TRAF6 | | Forward  Reverse | TACGATGTGGAGTTTGACCCA  CACTGCTTCCCGTAAAGCCAT |
| Arg-1 | | Forward  Reverse | CTCCAAGCCAAAGTCCTTAGAG  GGAGCTGTCATTAGGGACATCA |
| CD86 | | Forward  Reverse | TGTTTCCGTGGAGACGCAAG  TTGAGCCTTTGTAAATGGGCA |
| iNOS | | Forward  Reverse | GTTCTCAGCCCAACAATACAAGA  GTGGACGGGTCGATGTCAC |
| ACTA2 | | Forward  Reverse | GTCCCAGACATCAGGGAGTAA  TCGGATACTTCAGCGTCAGGA |
| CTGF | | Forward  Reverse | GGGCCTCTTCTGCGATTTC  ATCCAGGCAAGTGCATTGGTA |
| VEGF | | Forward  Reverse | CTGCCGTCCGATTGAGACC  CCCCTCCTTGTACCACTGTC |
| TGF-β | | Forward  Reverse | CTCCCGTGGCTTCTAGTGC  GCCTTAGTTTGGACAGGATCTG |
| Traf1 | | Forward  Reverse | AGGGTGGTGGAATTACAGCAA  GCAGTGTAGAAAGCTGGAGAG |
| Ccl4 | | Forward  Reverse | TTCCTGCTGTTTCTCTTACACCT  CTGTCTGCCTCTTTTGGTCAG |
| Cxcl2 | | Forward  Reverse | CCAACCACCAGGCTACAGG  GCGTCACACTCAAGCTCTG |
| Cxl10 | | Forward  Reverse | CCAAGTGCTGCCGTCATTTTC  GGCTCGCAGGGATGATTTCAA |
|  |  | |  |
